# Neuroscience of taste: unlocking the human taste code

**DOI:** 10.1186/s12868-024-00847-2

**Published:** 2024-03-21

**Authors:** Göran Hellekant

**Affiliations:** 1 School of Veterinary Medicine, Department of Biomedical Sciences, University of Wisconsin-Madison, 1656 Linden Drive, Madison, WI 53706 USA; 2 School of Medicine, Department of Biomedical Sciences, University of Minnesota Duluth Campus, 1035 University Drive, Duluth, MN 55812 USA; 3 School of Veterinary Medicine, Department of Animal Breeding and Genetics, Swedish University of Agricultural Sciences, Uppsala, Sweden

**Keywords:** Primate, Chimpanzee, Taste, Electrophysiology, Single fibers, Behavior, Phylogeny, Species differences, Labelled-line, Müller’s Law

## Abstract

Since antiquity human taste has been divided into 4–5 taste qualities. We realized in the early 1970s that taste qualities vary between species and that the sense of taste in species closer to humans such as primates should show a higher fidelity to human taste qualities than non-primates (Brouwer et al. in J Physiol 337:240, 1983). Here we present summary results of behavioral and single taste fiber recordings from the distant South American marmoset, through the Old World rhesus monkey to chimpanzee, the phylogenetically closest species to humans. Our data show that in these species taste is transmitted in labelled-lines to the CNS, so that when receptors on taste bud cells are stimulated, the cell sends action potentials through single taste nerve fibers to the CNS where they create taste, whose quality depends on the cortical area stimulated. In human, the taste qualites include, but are perhaps not limited to sweet, sour, salty, bitter and umami. Stimulation of cortical taste areas combined with inputs from internal organs, olfaction, vision, memory etc. leads to a choice to accept or reject intake of a compound. The labelled-line organization of taste is another example of Müller’s law of specific nerve energy, joining other somatic senses such as vision (Sperry in J Neurophysiol 8:15–28, 1945), olfaction (Ngai et al. in Cell 72:657–666, 1993), touch, temperature and pain to mention a few.

## Background

In science, as in many other areas where a researcher is faced with a large number of data points, it is necessary to be able to distinguish the “forest from the trees”. This is also the case with the question of how taste is coded. It is a topic whose solution is fraught with all kinds of confusing factors, such as species differences in food intake, influence of phylogeny, choice research methods, artifacts and inevitable variation of the data of behavioral and electrophysiological methods and technique chosen etc. All these factors, and several others, cover like “fog the terrain” that the researcher tries to penetrate to reveal the “reality”, whether it be features of the universe or the code of human taste.

The concept of taste qualities exists in every human culture [4, 5] and is generally used to describe the taste of any compound cf. [6]. It is easy to transfer these concepts from one human to another, which suggests that “taste quality” is universal in mankind. But does it exist in animals? Animals easily generalize a taste quality from one compound to another as shown by the extensive and reliable use of conditioned taste aversion methods, two-bottle preference comparison and other behavioral methods. Thus, there is no question that the amount of data overwhelmingly shows that this concept exists in many terrestral animals. The question then is if taste quality is based on some physiological or anatomical feature of the taste fibers in the chorda tympani (CT) and the glossopharyngeal (NG) nerves. Phrased differently, how can the nerve impulses resulting from the stimulation of lingual taste buds with, for example, sucrose, give rise to a taste quality? A third question is, what is included in a taste quality and is it the same in other species?

Attempts to relate the human taste qualities sweet, sour, bitter and salty with fiber type were made very early [7]. Zotterman proposed that activity in specific fibers codes for taste quality and called the fibers sweet, salty, sour and bitter fibers, although he found fibers that responded to stimuli which, from the human point of view, belonged to more than one taste quality.

An alternative explanation for the organization of taste was introduced by Pfaffmann [8]. In the late 1930s, Pfaffmann embarked on the mission: ‘‘to find objective evidence for the four basic taste sensitivities’’ by recording from single taste fibers [9]. He chose to study this in the cat, the most commonly used animal model at that time, and found little or no relationship between human taste qualities and taste fiber grouping; few or hardly any taste fibers responded to sugar, salt sensitive fibers responded to both NaCl and acids and bitter fibers responded not only to quinine but also to acids. Since he could not group the taste fibers according to human taste qualities, Pfaffmann introduced the term ‘‘across-fiber pattern’’ to describe the phenomenon [8]. In essence every taste fiber contributes to the taste of a stimulus in the across-fiber pattern. Expressed in a different way, the brain reads the incoming activity across all taste fibers. What was not understood at the time was, that instead of the four or five basic taste qualities that make up the human taste world, the gustatory world of the cat is dominated by amino acids found in meat [10]. Cats are insensitive to sugar.

The strongest support for the across-fiber pattern was probably published by Erickson who applied information from audition and vision to single taste fiber data from rats. He concluded that: “These data support an across-fiber pattern theory for taste quality sensitivity. These patterns, which signal the quality of the taste stimulus, are developed across a great number of fibers” [11]. It states that even if a taste fiber may have its greatest sensitivity at some point in an array of stimuli, it will respond to any stimulus if the strength of that stimulus is strong enough. This idea of how taste is coded has maintained its support [6, 12–17], also discussed more recently [18].

Some support for the labelled-line pattern of taste organization was obtained in an early study involving one rhesus monkey [19]. They wrote: “we can say, that this animal has a range of chemical sensitivity in its tongue which seems to agree with what one would expect from an animal phylogenetically so close to man”. Data suggesting some relationship between human taste qualities and monkey fiber groupings were published more than a decade later by Sato et al. [20]. Sato et al. later reanalyzed the data [21–23]. They classified their data according to the best stimulus criteria, but their data exerted minor influence on the scientific community who recorded from rodents and other non-primates. Consequently, based on the difficulties of relating taste qualities of humans with fiber types in laboratory species, the competing across-fiber pattern gained favor.

Meanwhile, studies continued to be published, which showed there is a selectivity among taste fibers, although the selectivity only partly may conform with human taste qualities. Hence, Frank coined the expression “best-fiber” selectivity based on data from hamsters [24] and the taste fibers were grouped according to their best stimulus: salt best, if response to NaCl was larger than to any other stimulus from all taste qualites, sweet-best if the response to sweet was largest etc. The use of this classification was easy when only one representative of each taste quality, e.g., sucrose, was used, but the interpretation became difficult with more than one representative of a taste quality. If for example, the order in size between the responses in a fiber was saccharin > QHCl (quinine hydrochloride) > sucrose, should the fiber be classified as a sweet-best or bitter-best fiber?

In 1974 Pfaffmann published a study of 7 sweet and 7 sweet-salt fibers from squirrel monkey that ‘‘not only does the best stimulus satisfactorily classify those sugar responding units, it also signifies that information (sweetness) is being carried by the fiber class. Furthermore, the concordance of order of effectiveness or sugar best fibers with the behavioral order leads us to conclude that this class is determining the behavior both in preference and reinforcement. The salt system does not seem to be motivating or stimulating such behavior.’’ [25]. He finished with: “We think the peaks define labelled-line clusters within each class, but that across-fiber patterning provides spectra a stimulation that may signal subtle differences or nuances within different taste classes. There is, therefore, both labelled-line coding and across-fiber patterning."

However, to my laboratory the importance of phylogenetic differences became more and more evident as we increased the number of animal species we recorded from. For example, the protein sweeteners monellin and thaumatin, which to human are several thousand times sweeter than sucrose gave no CT responses in guinea pig and rat, while they stimulated the CT nerve in *Cercopithecus aethiop*s, an Old World monkey [26]. Miraculin, from *Synsepalum dulcificum*, represents another example of phylogenetic differences. In human, miraculin adds sweet taste to sour stimuli. We used it in two Old-World monkeys*, Cercopithecus aethiop*s and *M. fascicularis* and recorded a doubling of the CT responses to acids after miraculin. Another example of species’ differences in taste is gymnemic acid from *Gymnema sylvestre* (GA) which has no effect on sweet in monkeys [27] while recordings of human CT show a complete loss of sweet taste after GA [28]. A later study added CT recordings in dog, hamster, pig and rabbit to the species which don’t taste the sweetness of monellin and thaumatin [29] and exhibited no taste modifying effect of miraculin and GA [30]. All these studies reinforced the importance of the phylogeny in taste.

**Fig. 1:**
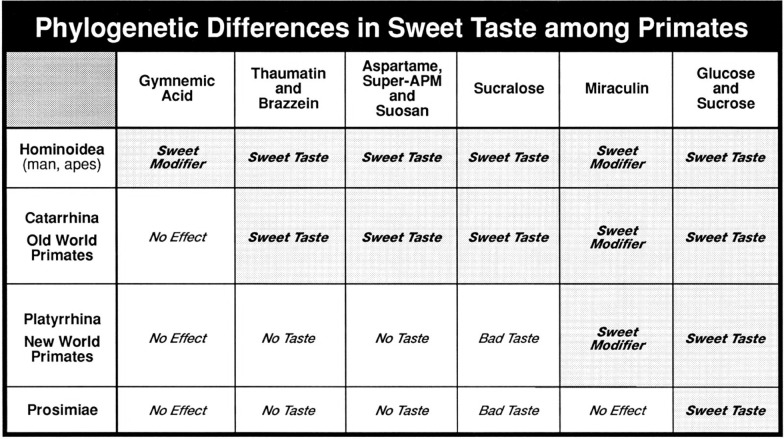
The table is a schematic demonstration of phylogenetic influences on sweet taste in primates. Compounds, which are sweet to human or exert an effect on human sweet taste, were plotted against 4 major divisions of primates in apes, Old World, New World and half monkeys. GH unpublished

Further examples are CT recordings and behavioral studies in a New World monkey, *Saguinus midas tamarin,* which showed the taste modifying effect of miraculin, but no sweetness of monellin and thaumatin [31]. In a large behavioral and electrophysiological overview of the sweetness of thaumatin and monellin in primates, we confirmed that in New World monkeys they are not sweet, while in Old World primates they are very sweet [32]. A final recording of the whole CT in 6 primates confirmed the miraculin effect in New World monkeys and the lack of an effect in Prosimian primates [33]. The table in Fig. 1 summarizes the above results in primates.

All of our above electrophysiological recordings were of whole taste nerves. Consequently, they could not shed light on how taste is coded in single taste fibers. However, they gave a good background to species differences and of the influence of phylogeny in taste. We realized that single fiber recordings were necessary if we wanted to solve the question of how taste is coded in human. A great help in this project was the publication by Smith and Frank, [24]. They formulated two criteria which should apply to the labelled-line pattern of coding taste in human: “*First, it must be demonstrated that a cluster of taste fibers has a high specificity to tastants within one of the human taste qualities and information in this particular cluster should be sufficient to distinguish stimuli of one taste quality from the others. Second, blockage of activity in a particular cluster must cause blockage of one taste quality, but of no others. Alternatively, onset of activity in a particular cluster must give rise to a taste quality. “*

As will be shown in the following, our studies in higher primates fulfill Smith’s and Frank’s criteria [24]. Their criteria for the labelled-line hypotheses together with the effects on taste by miraculin [1] and later GA gave us the tools we needed to pursue how taste is coded. We combined non-invasive and invasive methods. Among the non-invasive methods we used two-bottle preference for measurement of effects of miraculin, Mir, in monkeys. When determining the effect of gymnemic acid, GA, in chimpanzee we used a 6 point scale [34]. In the 1960s we developed a ventral surgical approach to expose the chorda tympani, CT, and glossopharyngeal, NG, nerves. The ventral surgical approach ensured that instead of sacrifice, the animals recovered without permanent deficits. We mention this to assure the reader that such results can be achieved routinely also in non-human primates through improvement of technique.

**Fig. 2:**
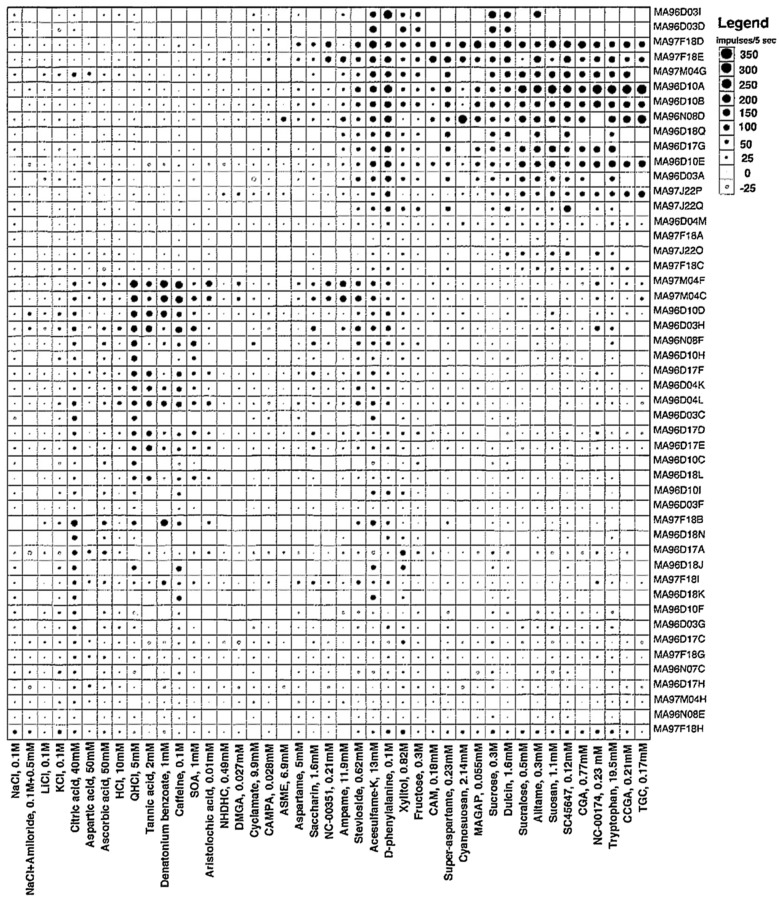
An overview of the response profiles of 49 single chorda tympani, CT, taste fibers of marmosets. The area of the circle represents impulse activity over the first five seconds of stimulation with impulse activity deducted during the first five seconds before stimulation. The size of the dots shows the intensity of a response. Open circles represent inhibition or absence of a response. Absence of a mark shows that data are missing. The stimuli were arranged along the X-axis in order of salty, sour, bitter and sweet. The fibers were arranged along the y axis in groups: NaCl, citric acid, QHCl and sucrose-best fibers. Note that there is no overlap between the fibers responding to bitter or sweet. We use the same topographical method in all overviews. [58]

**Fig. 3:**
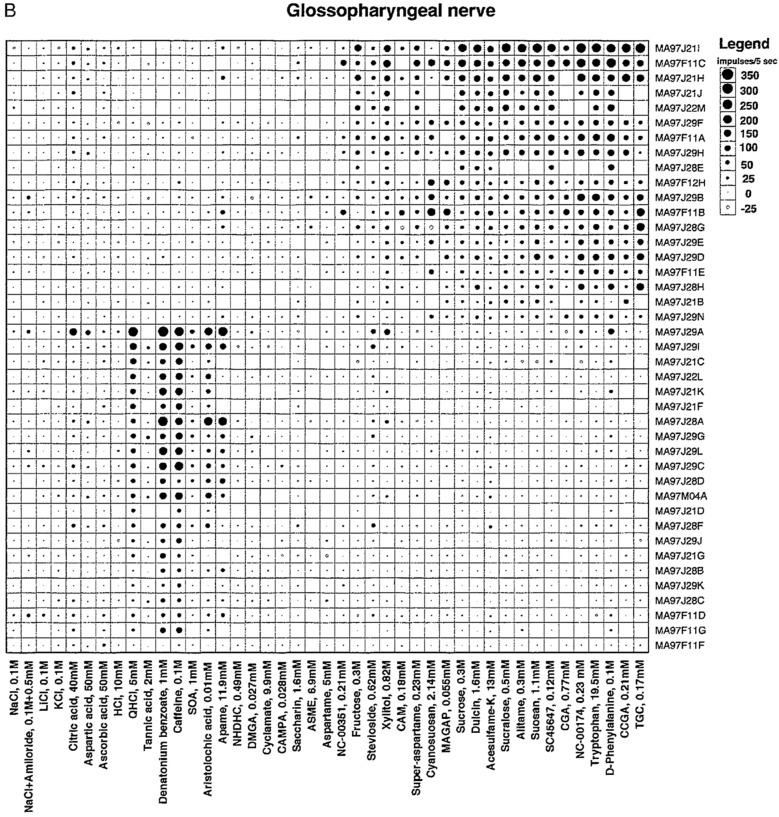
The plot presents a similar overview of 41 NG fibers. The overview shows no or small responses to NaCl, a significant response to bitter stimuli and a more intense response to the sweeteners in the NG nerve compared to responses in the CT. Note that there is no overlap between the fibers responding to bitter or sweet, neither in the CT nor the NG. [58]

**Fig. 4:**
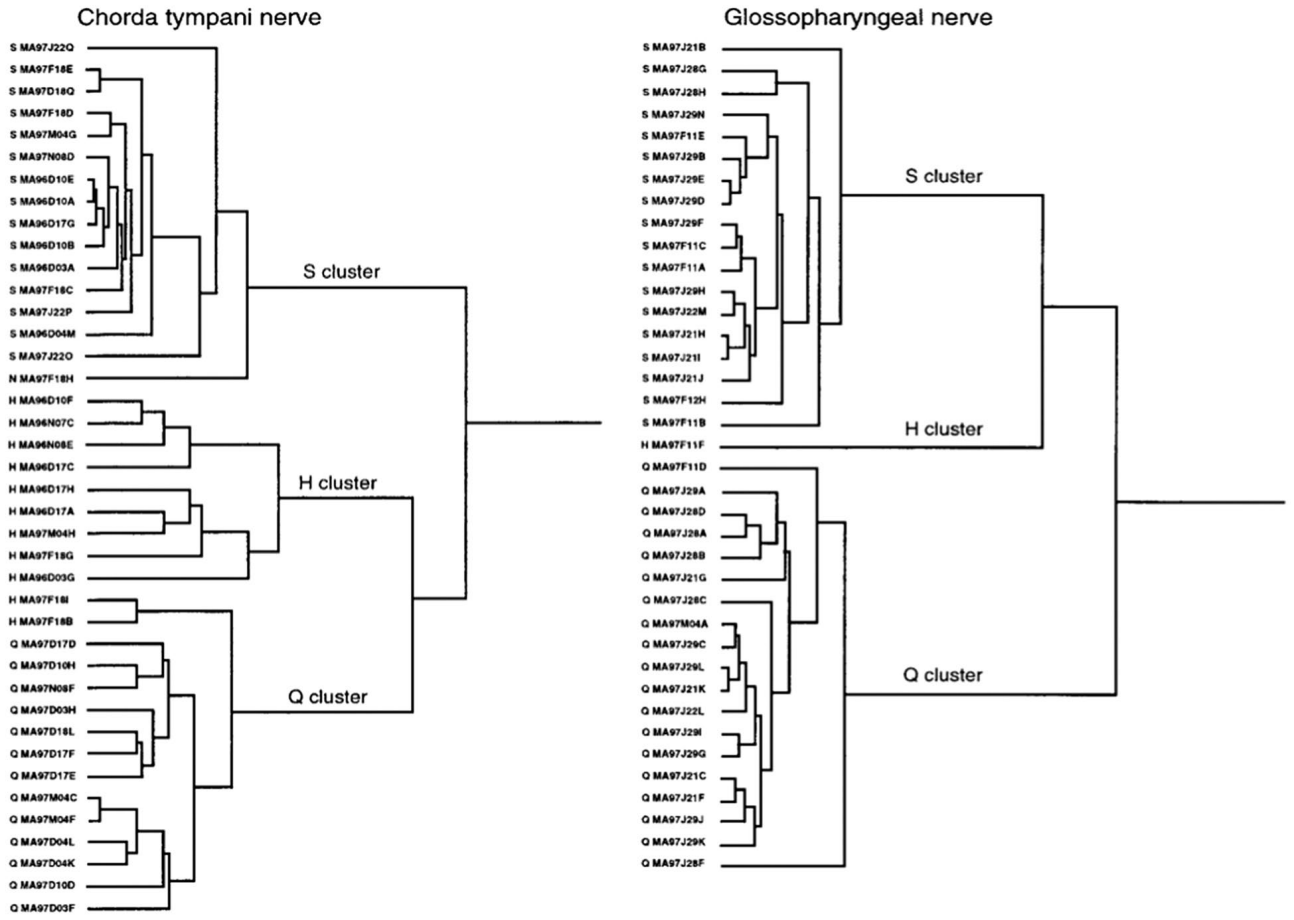
Shows the result of hierarchical cluster analysis of 40 CT fibers on the left side and 38 NG fibers on the right side. Listed on the left side of the dendrogram is each fiber’s number and the response category based on the best response to the four standard stimuli. NaCl (N), citric acid (H), quinine hydrochloride (Q) and sucrose (S) [58]

With regard to the electrophysiological recordings, we used two methods: either single fiber or multifiber recordings. For details see [35, 36]. We present results of single fiber recordings using a topographical method (examples are Figs. 2, 3, 12, 13, 23). To identify clusters of taste fibers in both CT and NG nerves we used hierarchical cluster analysis, which is a multivariate procedure for detecting natural grouping of the number of fibers with the number of stimuli. The results of hierarchical cluster analysis of the response profiles for taste nerve fibers are presented as a dendrogram (examples are Figs. 4, 14, 15, 24) in which the similarity of the response profiles determines how closely the fibers are positioned in the dendrogram. Based on the results one can determine how many clusters a single fiber population contain. The cluster categories in primates conform with the human taste qualities Q, N, H, S and M, so were labelled according to taste qualities they responded to: NaCl salty (N), MSG (M), sour (H), bitter (Q) and sweet (S). Non-primate sense of taste might also include some unknown cluster which is unrelated to the known taste categories as they are defined by human taste as we discovered in cow and pig. [37, 38].

Multidimensional Scaling, computes cordinates of points in a multidimensional space where each point represents the response to a particular stimulus. The results are plotted in a 2-D or 3-D space. It is a spatial presentation of the similarity between the stimuli using the response in each single fiber (examples are presented in Figs. 10, 29). The distance between the points shows how similar the taste of a compound is in relation to the taste of the other compounds from the animal’s point of view.

### Main text

This review will include single taste fibers from three non-human primates. It will begin with a South American monkey and continue with the Old World rhesus monkey and end with chimpanzee because it is the primate closest to human. The data presented in this overview are collected from 50 marmosets *Callithrix j. jacchus*, [33, 39–41], > 50 rhesus monkeys *Macaca mulatta* [1, 42–50] and 19 chimpanzees *Pan troglodytes* [34, 45, 51–58].

### *Callithrix j. jacchus* (Marmoset)

Our data and conclusions in this section are based on 6 studies [39–41, 58–60].

*Callithrix jacchus jacchus* is a small New World monkey belonging to the infraorder platyrrhina, which shared ancestry millions of years ago with the catarrhina group to which humans, apes and Old World monkeys belong. Since then, all platyrrhina and catarrhina species have undergone a great deal of differentiation so that extant primates of these two suborders differ in many aspects [61]. *C. jacchus* is a member of the *Callitrichidae* family. This family is among the most omnivorous or opportunistic feeders of living primates. Its normal diet consists of large amounts of fruit, leaves, buds, blossoms, green shoots, tree sap, and gums chewed from the bark of twigs and a high percentage of insects and small vertebrates, including eggs. Marmosets, like many other arboreal animals, relish the sweet sap or gum produced by trees. They will gnaw on bark, strip or bite off twigs and chew on them. These constituents often contain a high level of tannins, which have a bitter or astringent taste to humans. Thus, marmosets can be characterized as omnivorous with a very diverse diet.

#### We find that the taste fibers in marmosets fall into human categories

The topographical plots of single fibers response frequency demonstrate that, even in a primate relatively distant to human, tastants fall into human taste qualities (Figs. 2, 3**)**. In the CT, and less in the NG, there were large groups of S fibers which generally responded to all sweeteners, including acesulfame-K, D-phenylalanine, stevioside and xylitol. These last 4 sweeteners stimulated also Q fibers in both nerves which probably explains why behavioral experiments show their sweetness is less pleasant than carbohydrate sweeteners.

#### Hierarchical cluster analyses divide the taste fibers in both CT and NG into human taste qualities

Figure 4 shows three clusters of S, H and Q fibers on the anterior and posterior part of the tongue. These results suggest that marmoset shares 3 of the 5 human taste qualities.

#### Marmoset H, Q and S taste fibers respond to stimuli from more than one of the human taste qualities

**Fig. 5:**
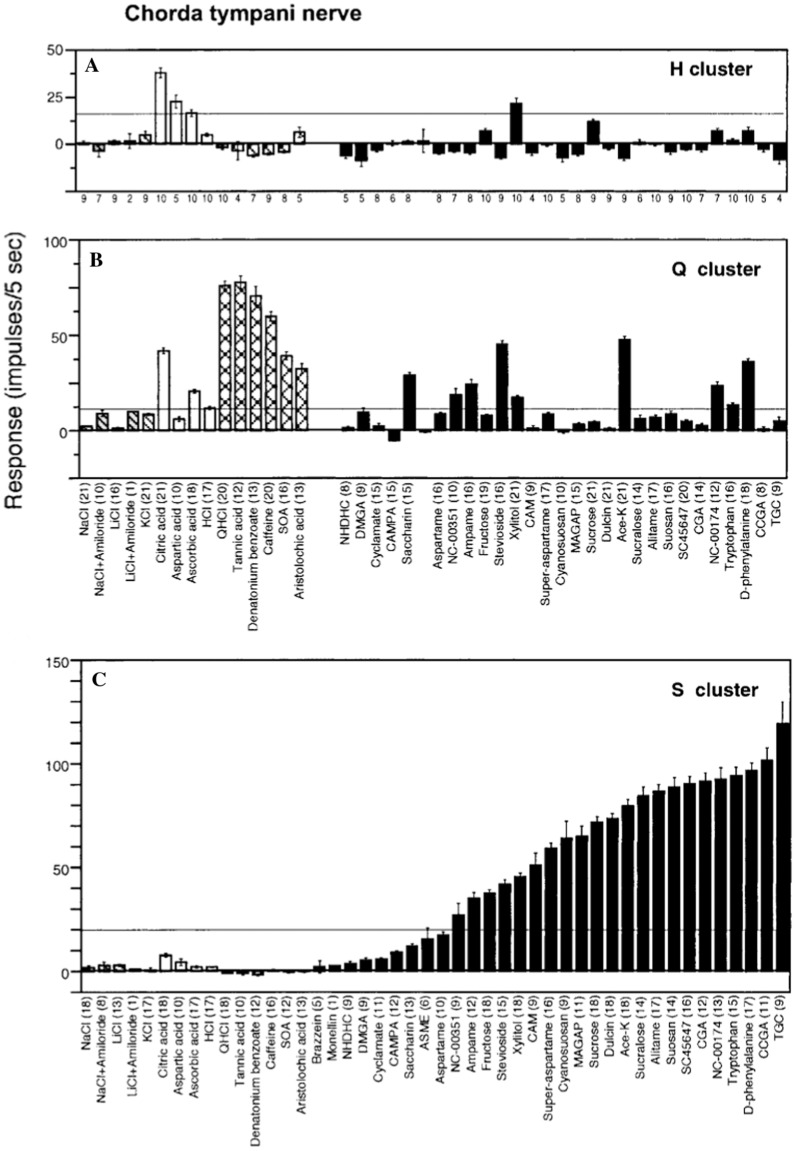
Shows the average response profiles of the 3 different taste clusters identified in the CT. The numbers below each column show the number of fibers which produced each response. The error bars show the standard error and the horizontal line drawn in each plot represents two standard deviations, SD, of the average spontaneous activity [58]

**Fig. 6:**
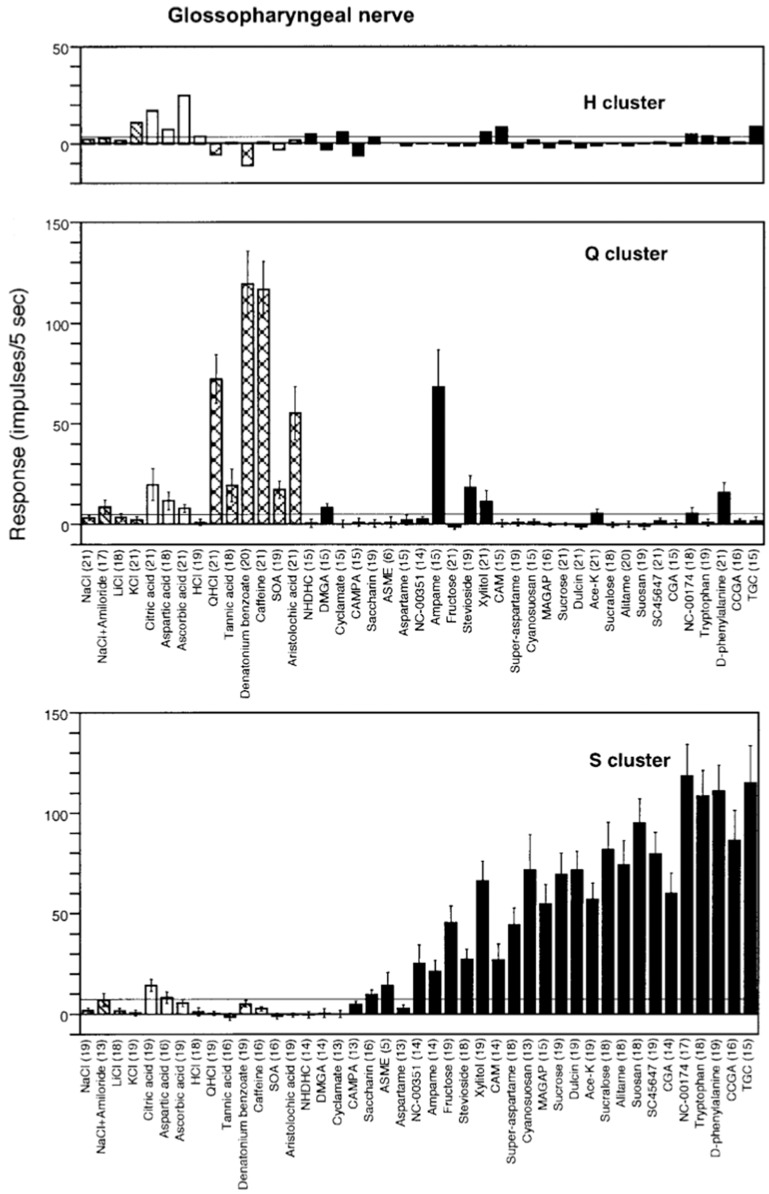
The average response profiles of the different taste clusters in the NG of marmosets arranged as in Fig. 5 [58]

The plots in Figs. 5, 6 show the average responses to each stimulus in the three clusters in the CT and NG. The intensity of the S fiber responses were similar but slightly lower than in the NG Q clusters. It is possible that to marmoset some compounds taste both sweet and bitter. However, taken together, these Marmoset data support and fulfill the first conditions for labelled-line coding*, namely that there are well-defined clusters of taste fibers linked to three human taste qualities.*

#### There are more H fibers on the front of the tongue and equal numbers of Q and S fibers on the back the back of the tonge

**Fig. 7:**
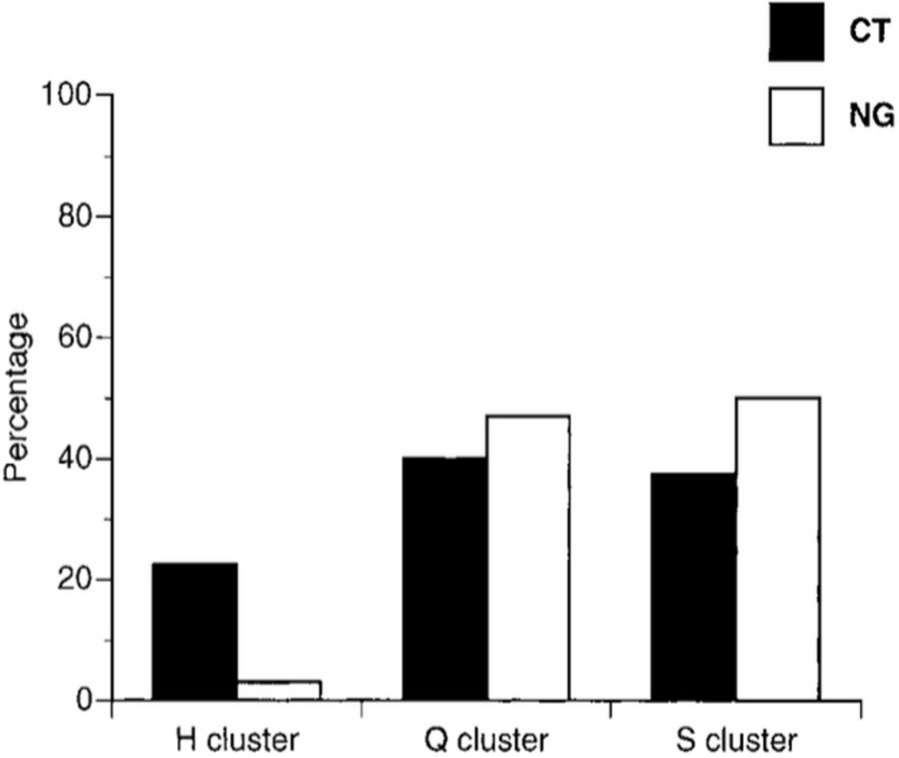
The figure shows the distribution of the different clusters in the CT (black columns) and NG (open columns). There is virtually no difference in number of Q and S fibers on anterior and posterior part of the tongue, but a large difference of H fibers in marmoset. [58]

Our data in Fig. 7 suggests that numbers of Q and S fibers were essentially the same in the CT and NG nerves, while there are more H fibers in the CT recordings compared to NG recordings. Both the overviews in Figs. 2, 3 and hierarchical cluster analyses in Fig. 4 show that 0.1 M NaCl did not stimulate any taste fibers in marmosets. This suggests that the ability to taste salt in its diet is not important to this species.

#### Miraculin in marmoset: the consumption of sour solutions more than doubled after miraculin exposure

**Fig. 8:**
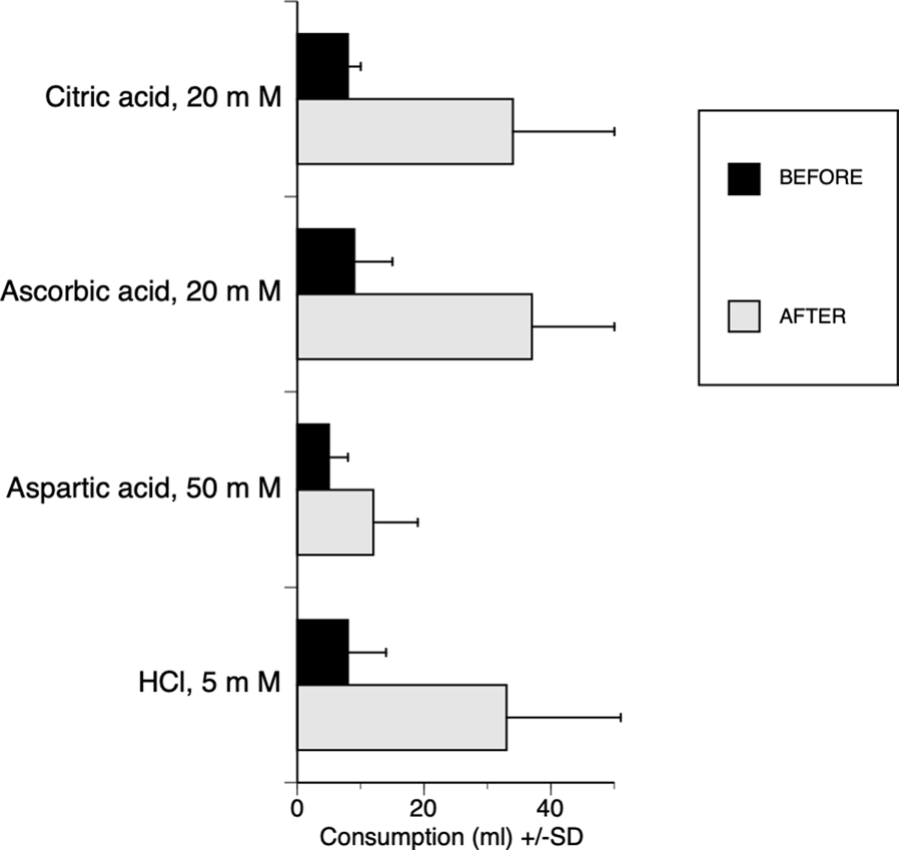
Shows the mean intake of water versus one of four acids (citric, ascorbic, aspartic and hydrochloric acid) to animals offered a choice of 50 ml water and 50 ml of acid, before (black) and after (gray) application of miraculin. Miraculin caused a significant increase in consumption of the acids. Redrawn from [41]

In humans, the sweetening effect on acids by miraculin from berries of *S. dulcificum* is well known and has been observed to change the sourness of a lemon so it tastes like a sweet orange. Figure 8 shows how monkeys consumed more acids after miraculin tongue exposure. For citric acid and ascorbic acid, the increased consumption was significant. The animals also consumed more of the aspartic and hydrochloric acids, although the increase was not significant. The question here is, can the labelled-line or across-fiber pattern explain this increase? Did miraculin increase the S fiber activity adding sweetness, or decreased the response of H fibers, thereby making the acids less sour or “did a change of the total assemble of taste nerve ensemble give rise to the added sweetness”? [11, 62].

#### Miraculin adds sweetness to acids by increasing S fiber activity

**Fig. 9:**
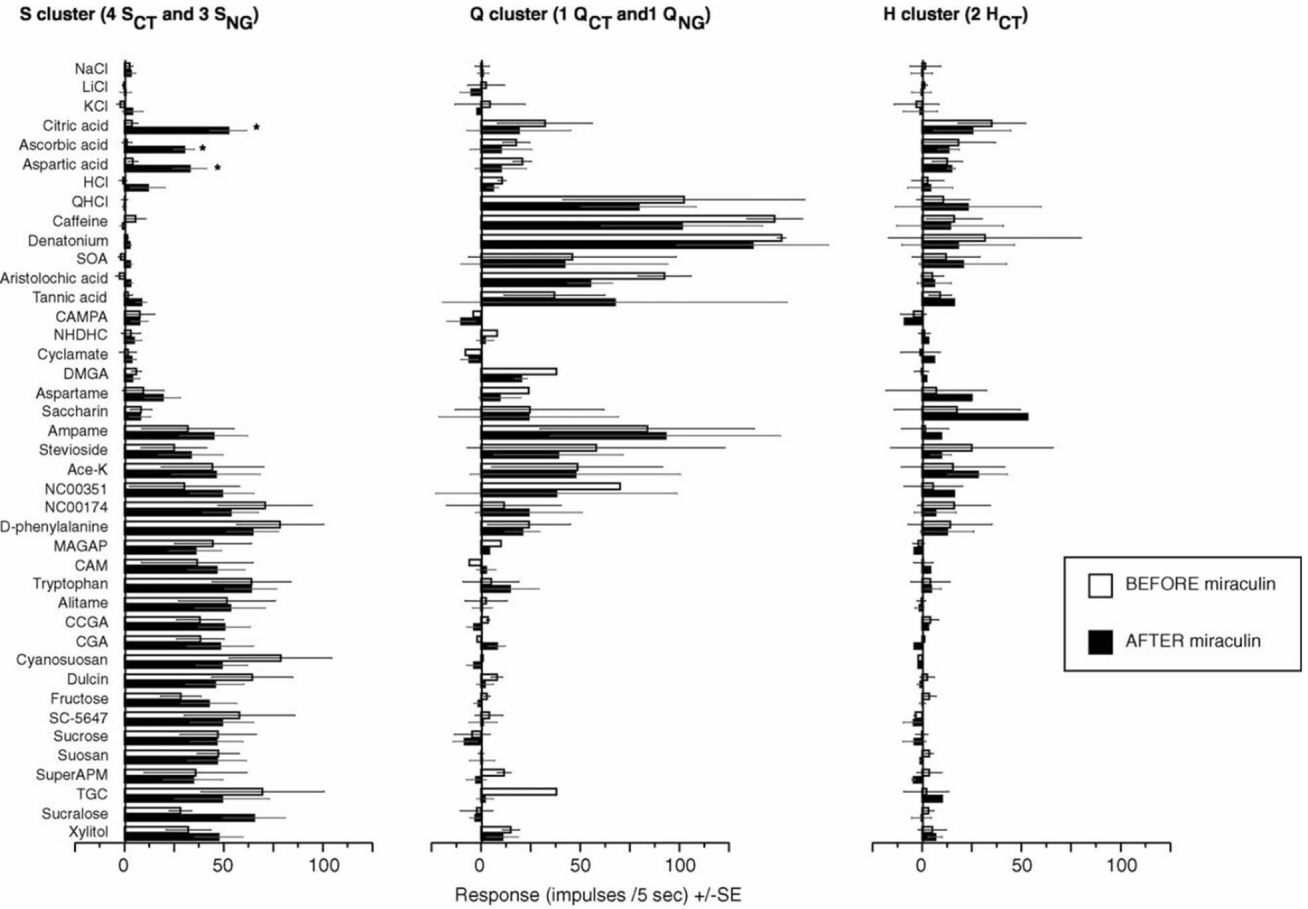
This figure shows the effect of miraculin on average responses of the combined S, Q and H fibers in the CT and NG nerves. The asterisks show that the only significant difference between the responses before and after miraculin was a larger response in the S fibers to the acids. This increased S fiber response is almost of the same magnitude as the response to sweet stimuli. Neither the Q nor the H fibers responses changed after miraculin. The plot shows an increased S fiber response that adds after miraculin, and effect on the H fiber activity, confirming the effect of miraculin is exclusively on the S fibers. This data clearly supports the labelled-line hypothesis [60]

As mentioned above (Fig. 4), cluster analysis identified 3 groups of fibers in the marmoset, corresponding to sweeteners (S cluster), bitter compounds (Q cluster) and acids (H cluster). We also recorded single nerve fiber responses to 41 different compounds (Fig. 9) and found significant differences between the responses before and after miraculin exposure, due to a large increase of the S fiber activities to the acids. This increased S response is almost of the same magnitude as their response to known sweet stimuli (astericks show statistical significance). Furthermore, there is no significant change of H fiber activity, confirming that the effect of miraculin is exclusively on S fibers. *This result clearly supports the labelled-line hypothesis, because the increase of action potentials occurred only in one cluster of taste fibers, namely the S fibers, which we know is the only group that increases intake.*

The basic mechanism by which miraculin functions is as following: Miraculin binds near or at the sweet receptor of TRCs. When sour compounds are placed on the tongue, oral pH is decreased which affects the structure of miraculin so that it stimulates the sweet receptor. Our explanation is supported by later studies [63]. Figures 8, 9 and 11 added to the explanation by showing that the stimulation of the sweet receptor increases impulse frequency in S fibers which is perceived as increased sweetness of acids.

#### Miraculin stimulates TRCs with S fibers adding sweetness to acids so that their consumption increases

**Fig. 10:**
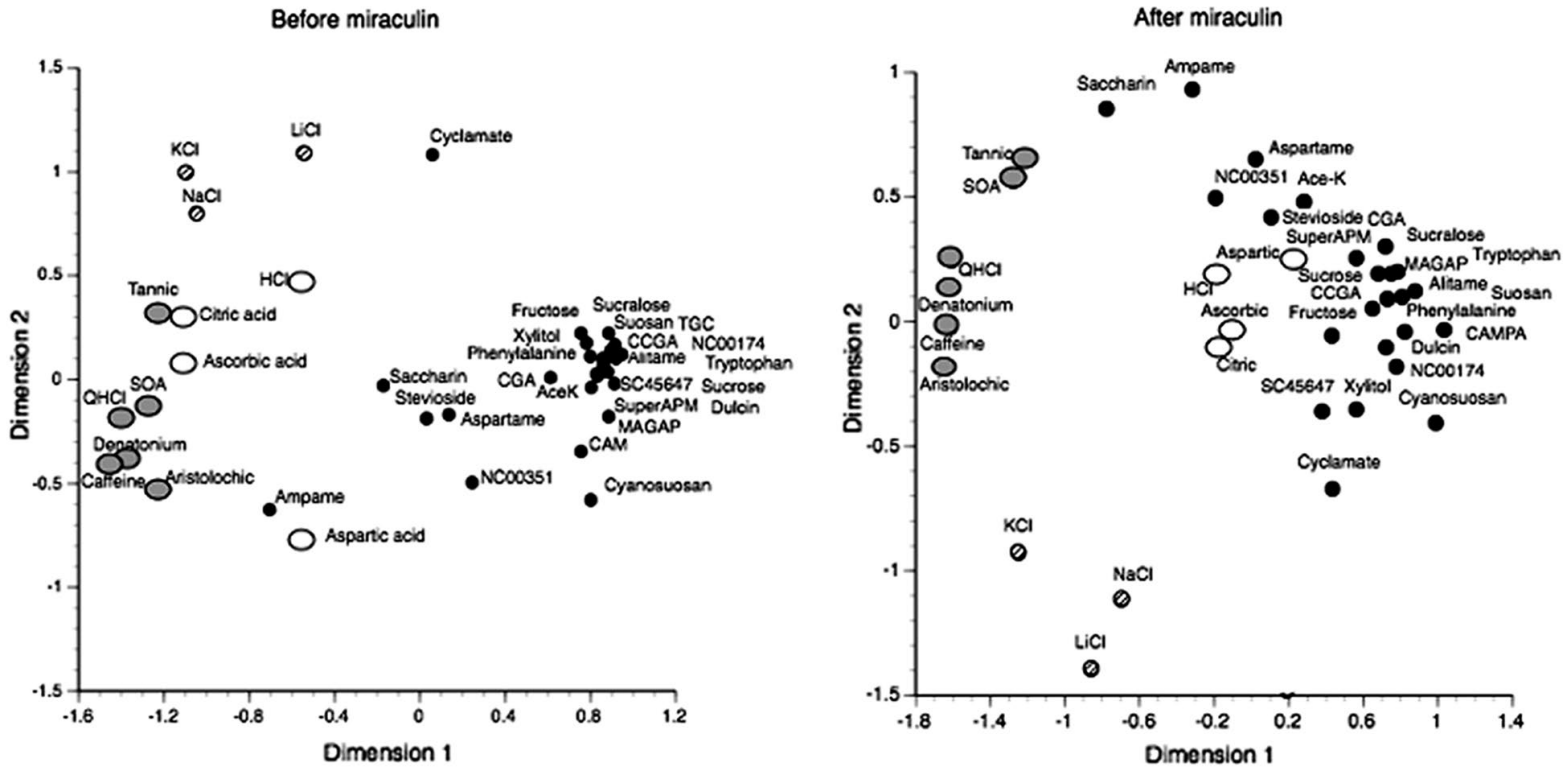
Distribution of taste stimuli in a two-dimensional space resulting from multidimensional scaling, MDS, before and after miraculin. The left plot shows results of MDS based data before miraculin application. Hatched symbols depict stimulation with salts, open symbols depict acids, gray symbols depict bitter and black circles depict sweeteners. The distance between the points indicates how similar the taste of a compound is in relation to the taste of other compounds. A large group of sweet fibers (dark circles) separated from the rest of the fibers and represent tastants liked by the marmosets. The right plot is data acquired after miraculin. It shows that the acids (open circles) have moved to a position closer to the sweetness, which suggests that S fibers also responded to the acids. The positions of the salts and the bitter compounds remain distant from sweeteners. [60]

It is well documented that sweet compounds are liked and therefore consumed, while sour compounds are less attractive. In Fig. 10 we present results of MDS using single fiber CT (*n* = 7) and NG (*n* = 4) recordings before and after miraculin. The left plot shows that the responses to acids (open circles) constituted a separate cluster close to the ordinate together with the other non-sweet stimuli. The right plot shows that miraculin application resulted in an additional taste quality, similar to adding sucrose, because the response to acids (open circles) shifted towards that of traditional sweeteners (black circles). The only possible cause for this shift is that miraculin stimulates the S receptors of TRC synapsing S fibers whose action potentials then add sweetness to the acid.

#### The ratio between Q and S fiber determines intake and miraculin increases S fiber activity of citric acid

**Fig. 11:**
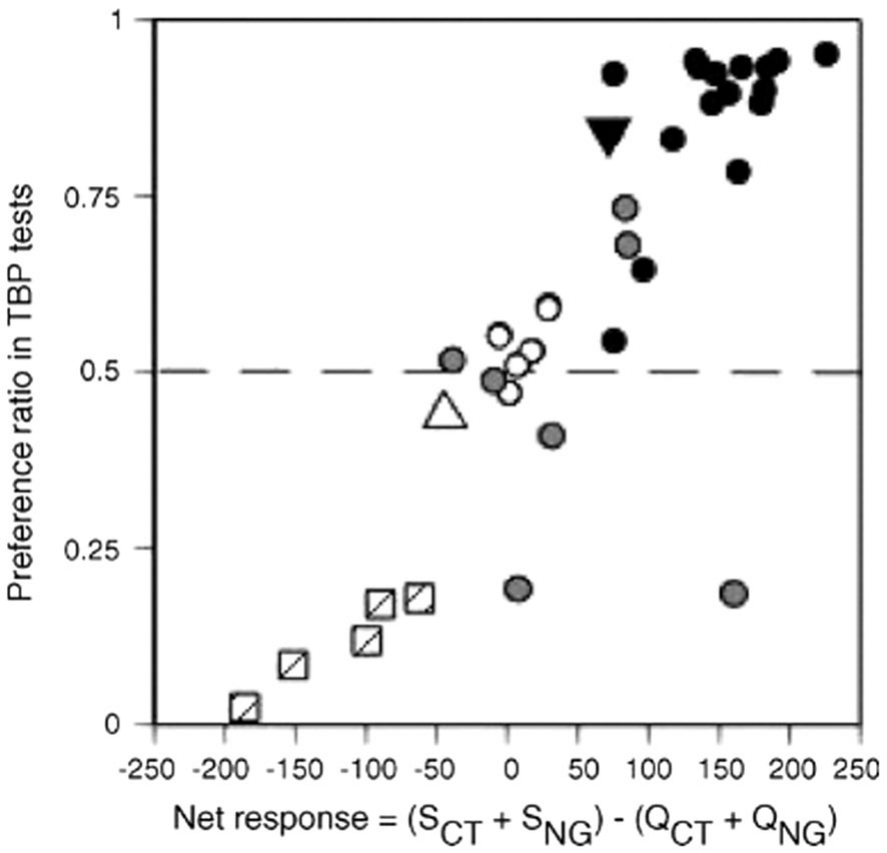
Relationship between preference ratios in behavioral experiments and Net responses in electrophysiological experiments. The Net response was calculated as (S_CT_ + S_NG_) − (Q_CT_ + Q_NG_). A linear relationship exists between preference and the Net response. The Spearman correlation coefficient for these two parameters was 0.85 (p < 0.01). Hatched squares indicate bitter stimuli (they stimulated only Q fibers); grey circles—sweeteners, which stimulated both Q and S fibers, black circles—sweeteners which stimulated only S fibers, white circles—sweeteners which did not stimulate any fibers. The open triangle depicts citric acid before miraculin and the inverted black triangle—citric acid after miraculin treatment [60]

We looked at the relationship between behavioral preferences in two-bottle preference tests and the Net responses to the tastants using fiber responses of both taste nerves in electrophysiological experiments [60]. Figure 11 suggests a strong correlation between preference ratios and Net nerve response. Thus, intake was high for compounds that stimulated only the S fibers and low for compounds that stimulated only the Q fibers. Intake of compounds with complex taste depends on the balance between the S and Q fiber responses. Figure 11 shows that after miraculin, citric acid response was moved toward the sweet compounds. Our interpretation is that citric acid after miraculin tasted more like a sweet compound than before. Therefore, miraculin added a sweet taste quality to the sour.

*In conclusion, the findings in marmosets satisfy the two conditions for labelled-line pattern of taste coding proposed by Smith and Frank *[24]*, namely “First, it must be demonstrated that a cluster of taste fibers has a high specificity to tastants within one of the human taste qualities and information in this particular cluster should be sufficient to distinguish stimuli of one taste quality from the others. Second, onset of activity in a particular cluster must give rise to a taste quality.”*

### *Macaca mulatta* (rhesus)

Here we summarize the results of several of our rhesus monkey studies [1, 42, 43, 46–48]. The rhesus monkey is an Old World primate, it is more closely related to humans than marmoset. For this reason, the rhesus monkey (*Macaca mulatta*) can be assumed to offer a more relevant model of human taste than marmosets. In the following, similarities and dissimilarities of the sense of taste of *M. mulatta* are presented.

#### Rhesus monkey has a more human-like distribution between the taste qualities on the front versus back of tongue compared to marmoset

**Fig. 12:**
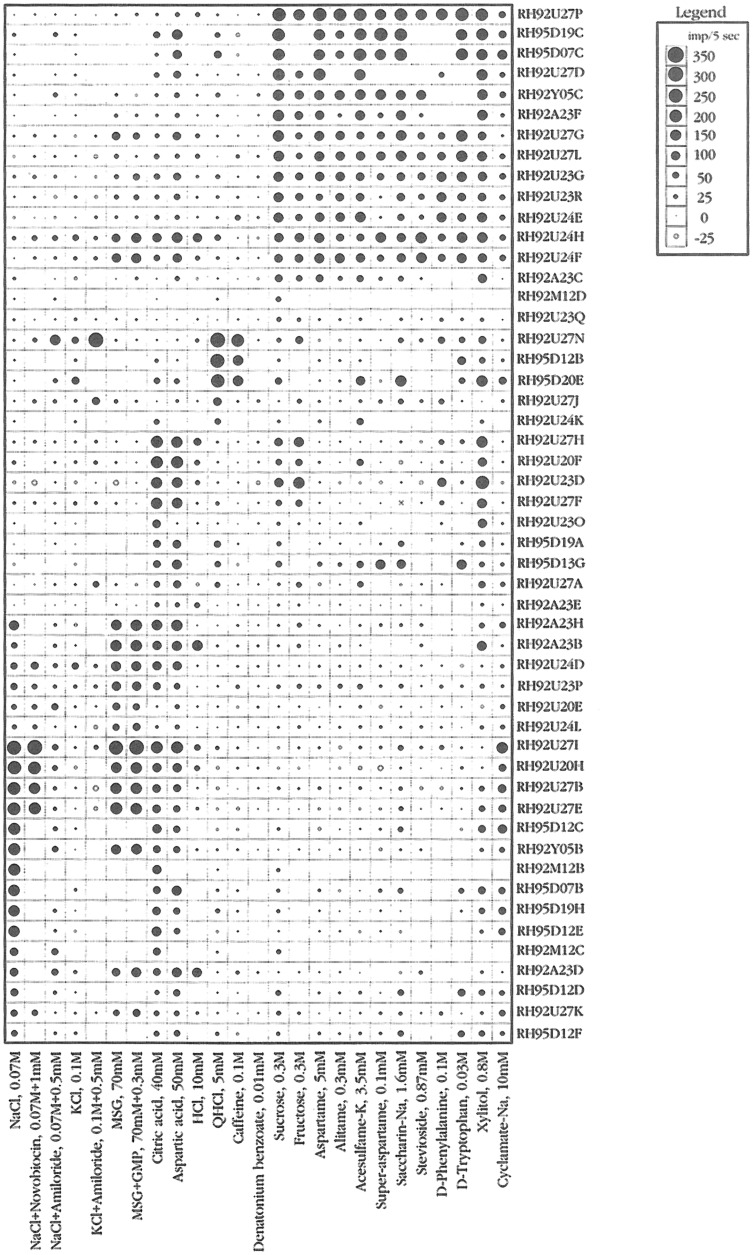
An overview from rhesus of the response profiles of 51 single CT fibers. Area of circles: impulse activity over the first 5 s of stimulation. Open circles: inhibition. Absence of a mark: data are missing. The stimuli were arranged along the X-axis in order of salt, sour, bitter, and sweet. The fibers were arranged along the Y-axis in groups: NaCl-, acid-, QHCl-, and sucrose-fibers. MSG, monosodium glutamate; GMP, guanosine 5’-monophosphate. [46]

**Fig. 13:**
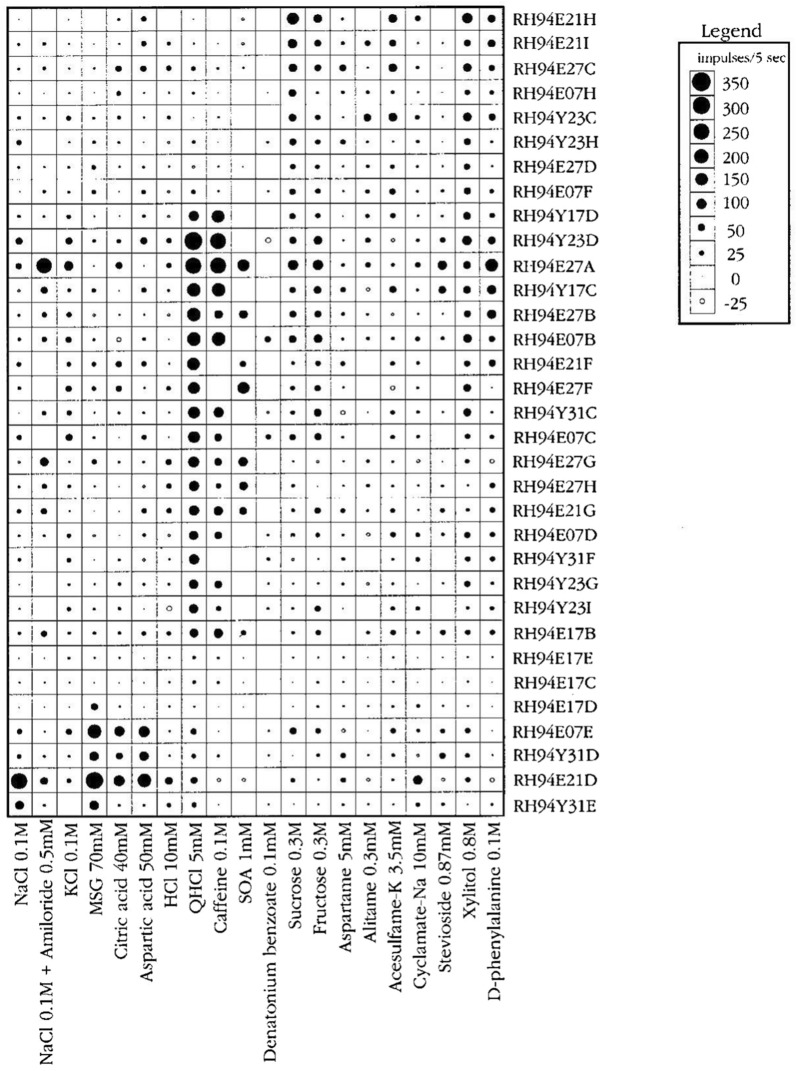
This figure presents a similar overview of the response profiles of 33 NG single fibers with the use of a topographical method as in Fig. 12. Area of circles: impulse activity over the first 5 s of stimulation. Open circles: inhibition. Absence of mark: data are missing. Arrangement of stimuli the same. Observe that salt, acids and sweet stimulates the anterior tongue while bitter the back mimicking the distribution in human. [46]

Figure 12 presents an overview of the responses in 51 CT fibers with the use of the topographical method and Fig. 13 gives a similar overview of the responses but of 33 fibers recorded in the NG. The most interesting observation is the large number of N, H and S fibers on the front and Q fibers on the back of the tongue. This is a similar distribution as taste qualities in human.

#### Hierarchical cluster analysis supports a human-like distribution of taste sensitivity

**Fig. 14:**
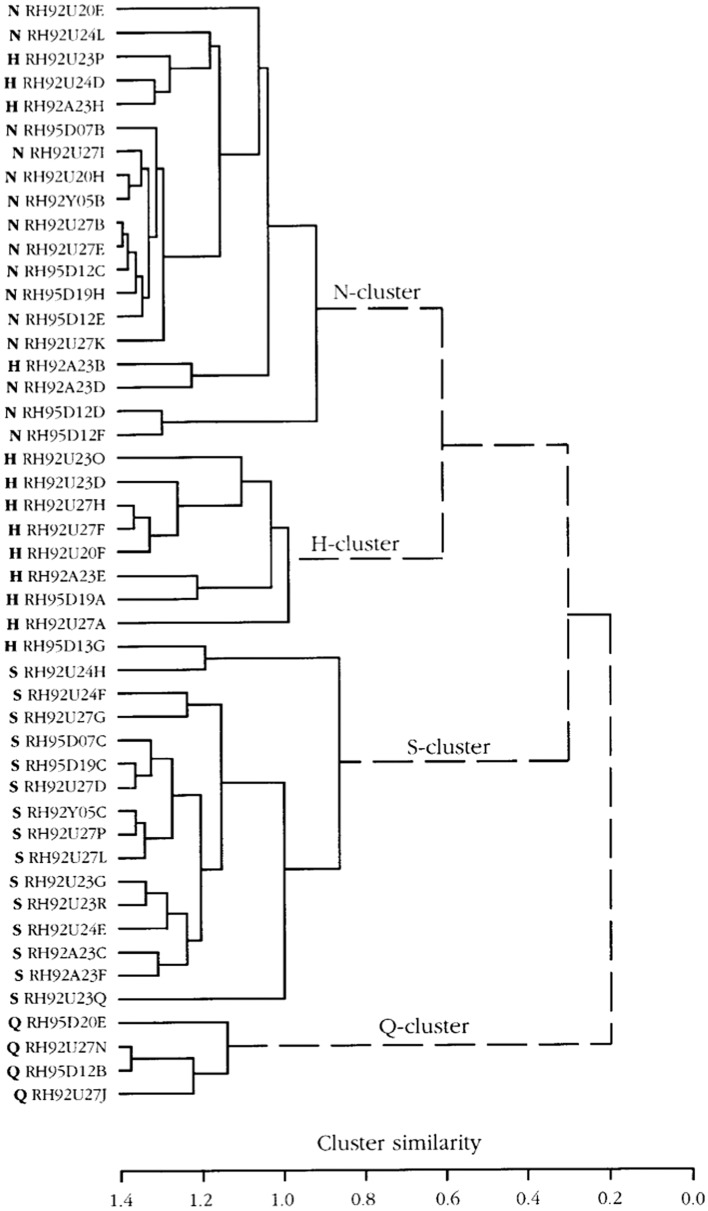
Hierarchical cluster analysis of the response profiles for 47 CT fibers. The identity and number of the fiber and response category on the basis of its best response to the basic solutions are listed on the left side of the dendrograms. Inter-cluster similarity was measured with the use of the Pearson correlation coefficient and cluster analysis proceeded according to the average linkage method. Fiber number and response category based on response to the 4 basic solutions of N, H, Q, and S: NaCl-, citric acid-, QHCl-, and sucrose fibers, respectively. [46]

**Fig. 15:**
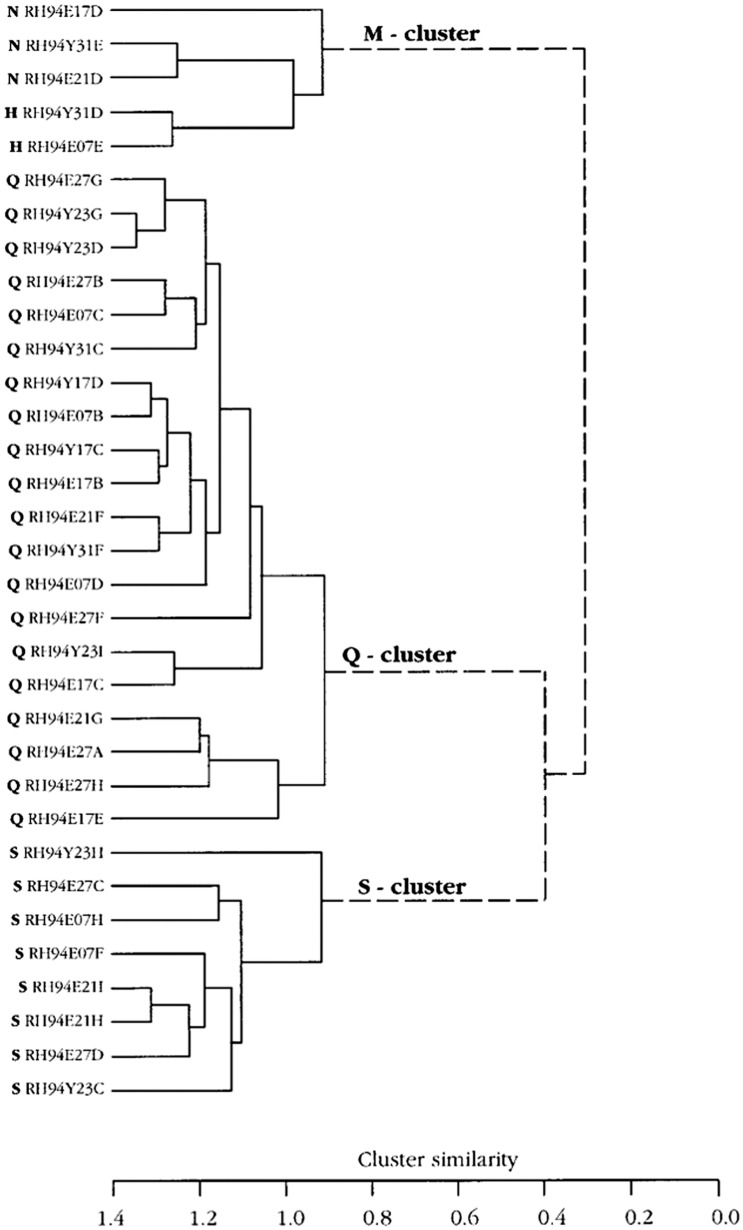
Hierarchical cluster analysis of the response profiles for 33 NG single fibers. Inter-cluster similarity was measured with the use of the Pearson correlation coefficient and cluster analysis proceeded according to the average linkage method. Fiber number and response category based on response to the 4 standard solutions of N, H, Q and S: NaCl-, citric acid-, QHCl-, and sucrose fibers, respectively. [46]

In Fig. 14**,** the CT analysis shows all four human taste qualities are represented: salt (N-cluster), sour (H-cluster), sweet (S-cluster) and bitter (Q-cluster). Many of the fibers responding to salt also responded to MSG, suggesting that MSG to rhesus may also taste salty, which probably is what most human unfamiliar with umami taste would call it. The cluster analysis of NG is shown in Fig. 15. and resulted in three major clusters: M, coinciding to the taste qualities of monosodium glutamate, and Q, and S clusters. The dendrogram stresses the relatively large number of Q fibers in the NG nerve compared to in the CT nerve and the lack of an H cluster and the emergence of an M cluster, which indicate a significant response to umami compounds. This agrees with data in humans where umami can be considered a taste quality, carried by a separate group of single fibers to CNS [52].

#### All human taste qualities exist in rhesus CT

**Fig. 16:**
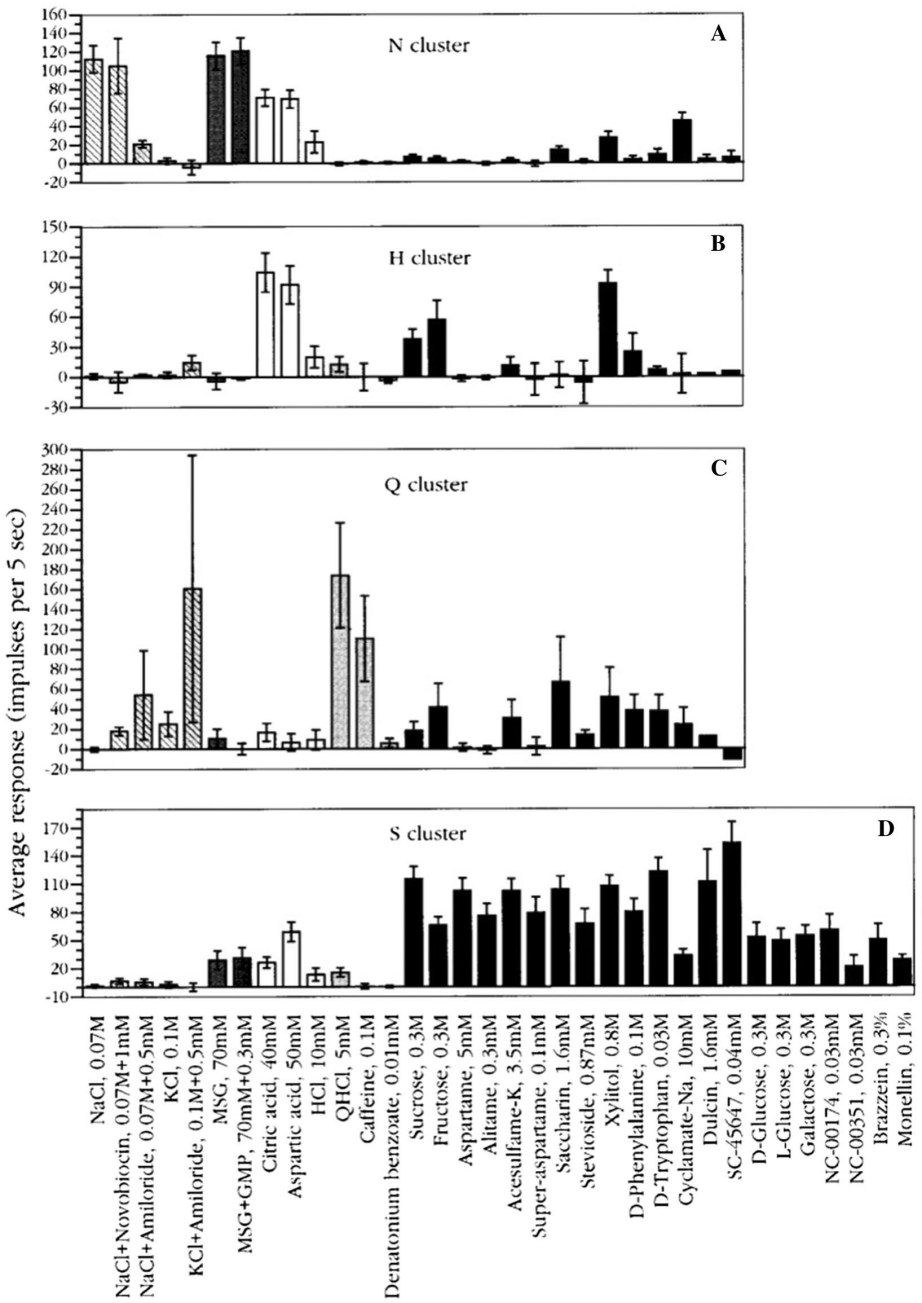
Average response profiles of 4 major clusters in rhesus CT fibers. The stimuli are listed along the X-axis, whereas the average impulse activity measured over 5 s is plotted along the Y-axis. Error bars indicate SE. Hatched columns: salts. Dark gray columns: umami compounds. Open columns: acids. Light gray columns: bitter compounds. Black columns: sweeteners. It is interesting that the cluster analsis added an H-clusters to the 3 other. [46]

The plot in Fig. 16 shows the average response profiles of four clusters identified in CT fibers. In the following we discuss each cluster.

N cluster: The N cluster included 19 fibers and was characterized by strong responses to NaCl and MSG alone or mixed with guanosine 5-monophosphate. Amiloride abolished the response to NaCl. KCl elicited no response in N fibers. These N fibers also did not respond to bitter compounds, and of the sweeteners only to sodium cyclamate and xylitol.

H cluster: Citric and aspartic acids elicited the best responses in H fibers. Generally, there was no response to any other stimulus. There was no response to salty and bitter compounds, indicating that, to rhesus, these compounds lacked sour taste.

Q cluster: This cluster consisted of four Q fibers which responded well to QHCl and caffeine but not to the bitter compound, 0.01 mM denatonium benzoate. Considering the fact that amiloride tastes bitter to humans, it is interesting that NaCl with amiloride and KCl with amiloride elicited a response in these Q fibers, suggesting a similar taste of these compounds to rhesus monkeys.

S cluster: The S cluster was the second largest cluster and consisted of 16 fibers and responded to every sweet stimulus. These included D- and L-glucose; galactose; dulcin; three guanidine derivatives: SC-45647, NC-00174, and NC-00351 in addition to the sweet protein brazzein and monellin. Earlier we found that S fibers in rhesus monkeys also respond to thaumatin, aspartame and acesulfame-k [1]. In fact, we have yet to see a compound that is sweet to humans that does not stimulate S fibers in the rhesus monkey [64]. This shows a closer phylogenetic relationship to human than in the marmoset, but less than in the chimpanzee as will be seen below.

**Fig. 17:**
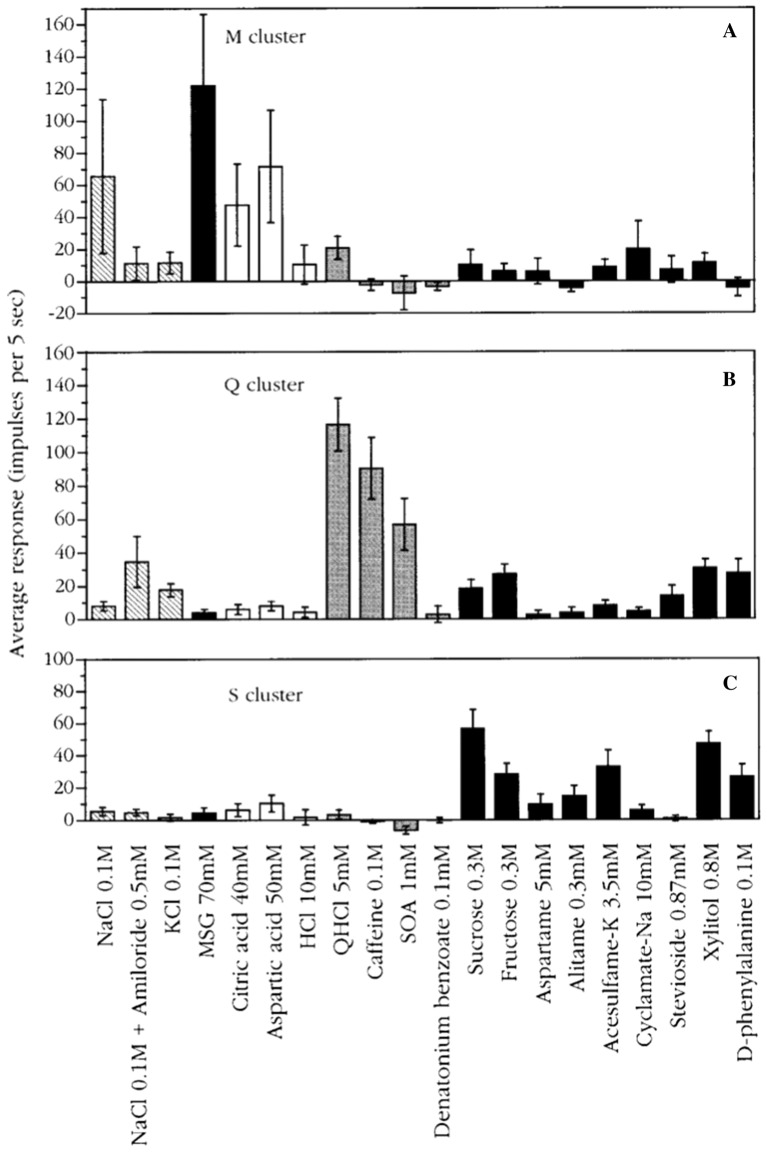
Average response profiles of 3 major clusters of rhesus NG fibers: M fiber cluster, Q fiber cluster, and S fiber cluster. Error bars: SE. Different patterns of columns indicate different taste qualities, as in Fig. 16. The stimuli are listed along the X-axis, whereas the average impulse activity measured over 5 s is plotted along the Y-axis. Error bars indicate SE. [46]

*Figure *17*Shows a similar distribution on the rhesus tongue as has been reported in human supporting the hypothesis of labelled-line in humans*.

The plot shows the average response profiles of three NG clusters in the same manner as for the CT nerve.

M cluster: A few fibers were identified as M fibers because MSG gave the largest response in this cluster. NaCl, citric acid, and aspartic acid elicited a substantial response whereas bitter compounds and sweeteners did not stimulate M fibers.

Q cluster: Q fibers responded mainly to three of the bitter compounds, but minimally to denatonium and constituted the largest group in NG of 20 fibers out of a total of 33. We added sucrose octa acetate (SOA), a compound bitter to humans, to our array of stimuli for the NG recordings. The data indicate that SOA stimulated these fibers as well as QHCl and caffeine. The lack of a response to denatonium benzoate corroborates our findings in the CT. The finding that NaCl with amiloride elicited a larger response in the Q cluster than in the M cluster corroborates the effects of NaCl with amiloride on the CT. The Q cluster did not respond to NaCl, acids, or most sweeteners, with the exceptions of sucrose, fructose, xylitol, and D-phenylalanine which elicited some response.

S cluster: The S fibers constituted the second largest cluster of eight fibers with a significant response to sucrose but its response to sweeteners was generally smaller and less significant than in the CT nerve. With regard to sweeteners, rhesus monkeys perceived the sweet taste of a larger number of sweeteners than marmoset. See Fig. 38. Among these are aspartame, generally used by humans, but also the super-sweet proteins monellin, thaumatin and brazzein [65–67]. This makes sense since these sweet proteins have not been found in the Americas and the rhesus monkey is an Old World primate.

**Fig. 18:**
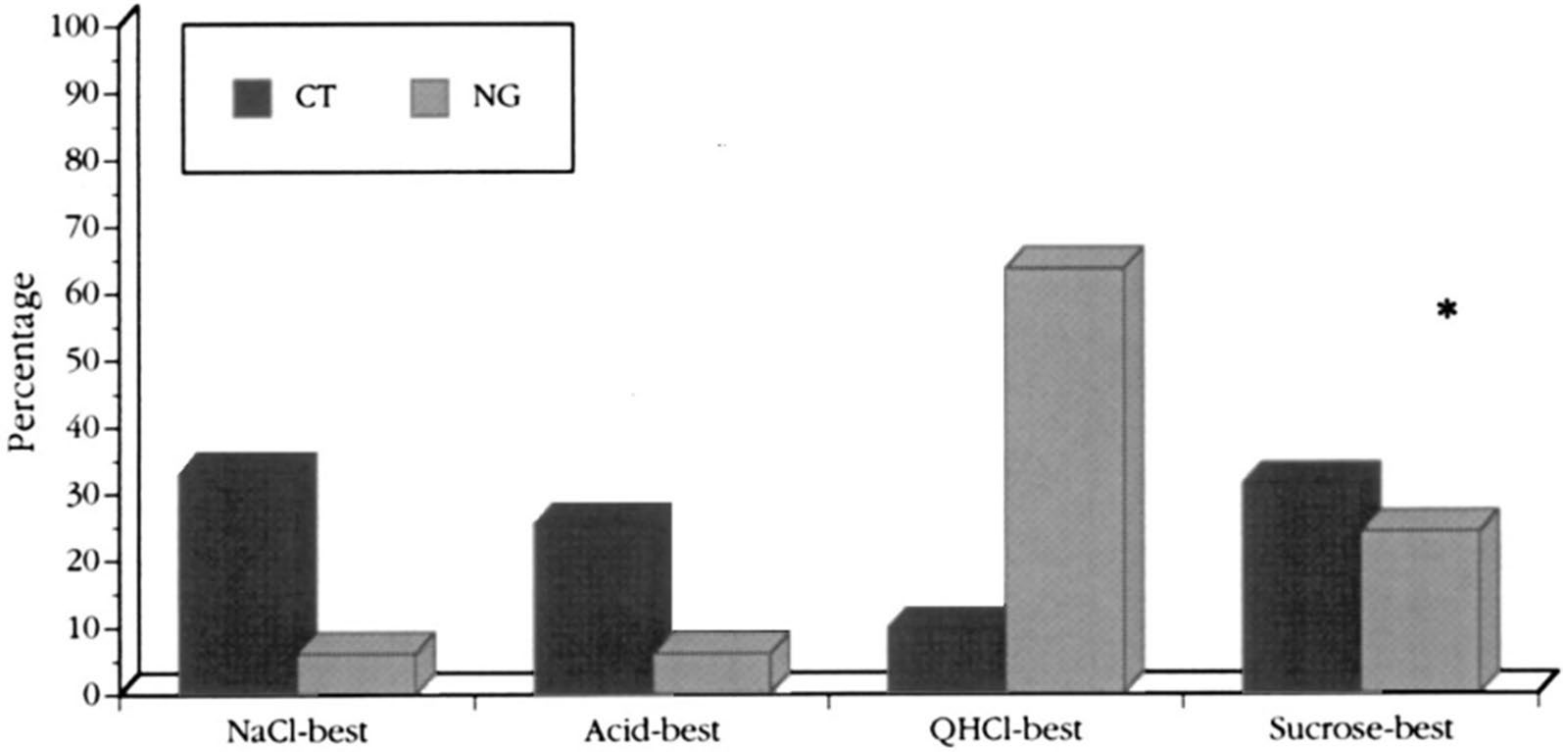
Distribution of 4 different types of single taste fibers according to their best response to the 4 basic stimuli in CT and NG. The most striking difference is a large number of CT fibers responding to NaCl, including umami compounds, while the Q fibers in the NG dominate. The asterisk over sucrose signifies the statistically significant difference between CT and NG in regard to responses to sucrose. This difference mimics and explains the human experience of tasting mostly salt and sweet on the front and bitter on the back of the tongue. [46]

Figure 18* shows that in rhesus the distribution of taste qualities over the tongue is similar to the distribution in human.*

The staples show the distribution of the 4 types of single type taste fibers in the CT and NG nerves. The most striking observation is a large number of CT fibers responding to NaCl, including umami compounds, while the Q fibers in the NG overwhelm. This is similar to the human experience of tasting mostly salty and sweet on the front and bitter on the back of the tongue. The asterisk over sucrose signifies the statistically significant difference between CT and NG in regard to responses to sucrose.

**Fig. 19:**
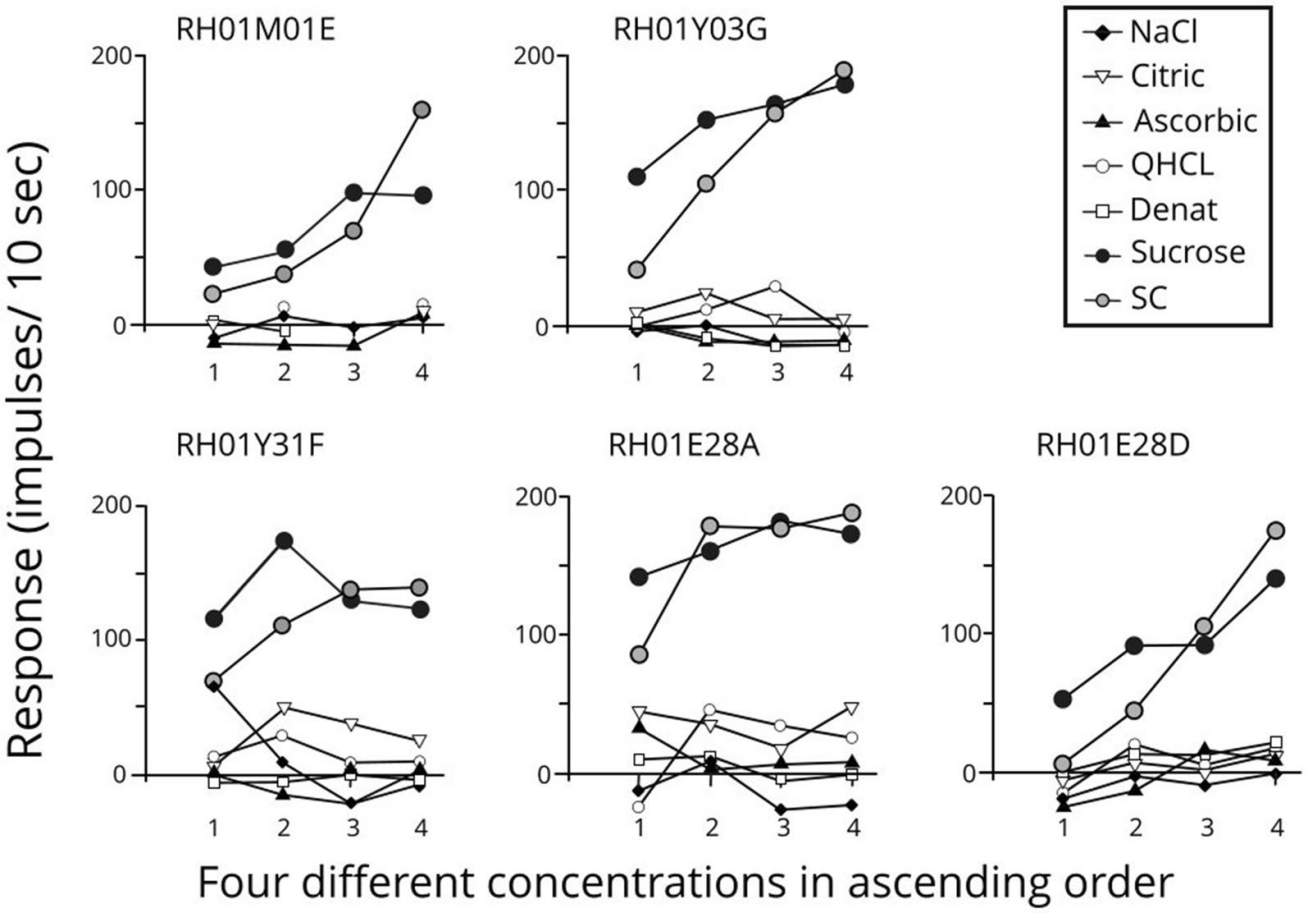
The average nerve impulses to taste stimuli in 5 N fibers, 5 S fibers, 5 Q fibers and 5 H fibers in which the responses of 4 different concentrations of 7 taste stimuli representing the 4 taste qualities are plotted. The concentrations for the different stimuli were: 0.05–0.2 M NaCl; 20–100 mM citric acid; 0–200 mM ascorbic acid; 1–30 mM QHCl; 0.2–2 mM denatonium benzoate; 0.1–0.8 M sucrose; and 0.01–1 mM SC45647. Thus, the concentration ranged at least 4 times and for some stimuli, 100 times between the lowest and highest used. It is evident that each fiber type maintained its response to the same taste quality throughout concentration changes. This would not be the case in across-fiber pattern coding but supports the labelled-line theory. GH unpublished

Figure 19* shows that all 4 fiber types respond only to stimuli within one taste quality.*

Advocates for the across-fiber pattern theory argue that any fiber will respond if the concentration of tastants is increased enough [11, 62]. An important condition for the validity of the labelled-line theory is that the taste quality that a fiber conveys is the same at all concentrations of its stimulus. Figure 19 addresses this question. It shows the average number of impulses to taste stimuli in 5 N fibers, 5 S fibers, 5 Q fibers and 5 H fibers in which the responses of 4 different concentrations of 7 taste stimuli representing the 4 taste qualities are plotted. Thus, the concentration ranged at least 4 times and for some stimuli 100 times between the lowest and highest used. In each fiber group there was only an increased response to stimuli within their own taste quality. For example, the only stimuli that increased S fiber activity were the artificial sweetener SC45647 and sucrose while all the other non-sweet compounds gave no response regardless of their concentration. These data represent strong support of the labelled-line theory.

**Fig. 20:**
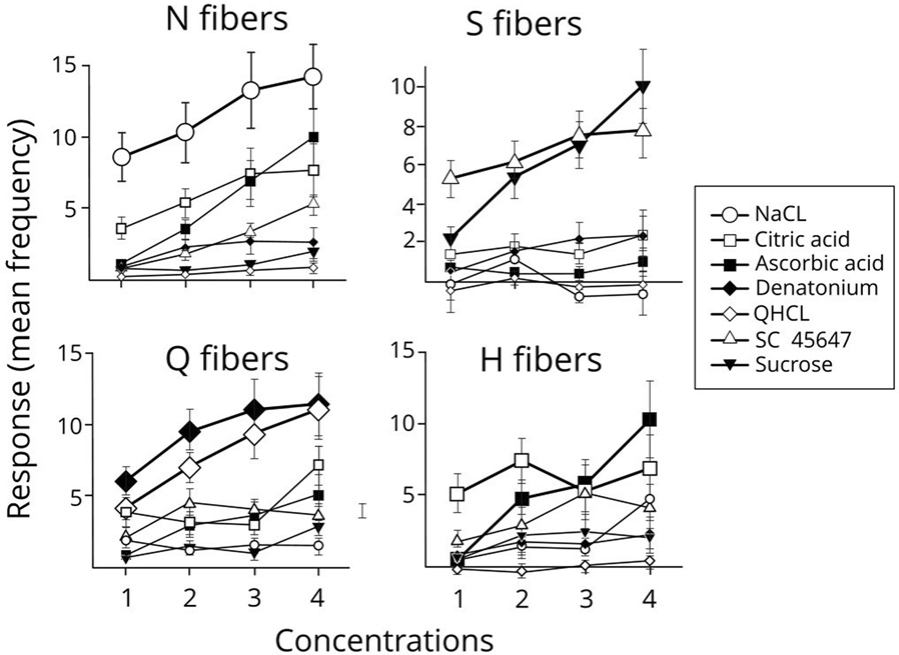
Responses of 5 S fibers were plotted at 4 concentrations for 7 different stimuli. The plot shows that the S fibers increased their responses to sweeteners but not to the other 5 stimuli; NaCl, citric and ascorbic acids, QHCl and denatonium This shows two important features. The S fibers respond exclusively to increase of sweetener concentrations and the increase of the non-sweeteners had no effect on the S fibers. Only sucrose and SC 45647 increased the frequency of action potentials in these 5 S fibers. The result does not support the across-fiber pattern theory but instead strongly supports the conclusion that labelled-line includes all taste fiber types, N, Q, H, S. GH unpublished

Figure 20* shows that change of concentrations of salt, acids, bitter or sweet did not change the classification of S fibers.*

According to the labelled-line theory each taste fiber mediates only one taste quality. Increased concentration of the adequate stimulus, for example, a sweet stimulus in a S fiber, should only elicit more action potentials in the S fiber, but not in a N, Q, H or M fiber. Similarly, responses in an S fiber should not be affected by non-sweet stimuli if taste is conveyed according to labelled-line theory. None of the tested S fibers responded in a different way.

*The general conclusion of these results is that each taste quality is represented by a cluster of taste fibers whose taste quality fidelity is maintained over changing concentrations of stimulus. These results support the validity of the labelled-line theory in taste coding.*

### Miraculin

**Fig. 21:**
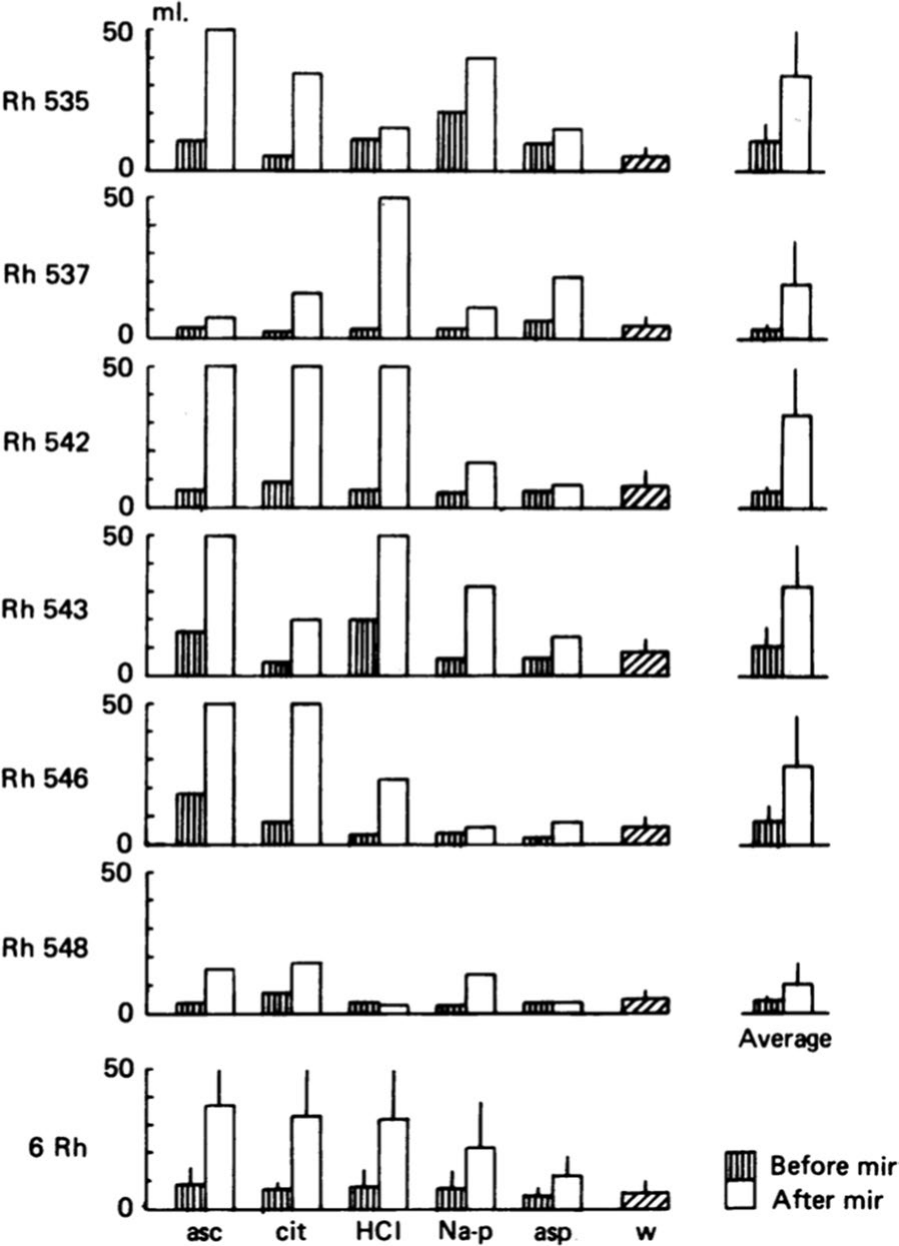
The intake of acid solution, offered as a choice with water in two-bottle preference tests, before and after miraculin (mir). Animals were presented with 50 ml of each acid and water. The acids were 0.02 M-ascorbic (asc), 0.02 M-citric (cit), 0.005 M-hydrochloric acid (HCl), 0.05 M-phosphate buffer (Na-p), 0.05 M- aspartic acid (asp) and water. Columns show intake before (hatched columns) and after (open columns) miraculin. The column labelled is the average water intake before and after miraculin. The average consumption of acids by each animal is shown on the right individual column. The average intake for each acid is shown at the bottom (error bars, 1 S.D.). The figure shows that miraculin increases intake of acids.

Figure 21 shows that a few mg of miraculin on the tongue more than doubles intake of acids.

These data sugget that rhesus monkeys experience a similar addition of sweetness in acids after miraculin as humans do.

**Fig. 22:**
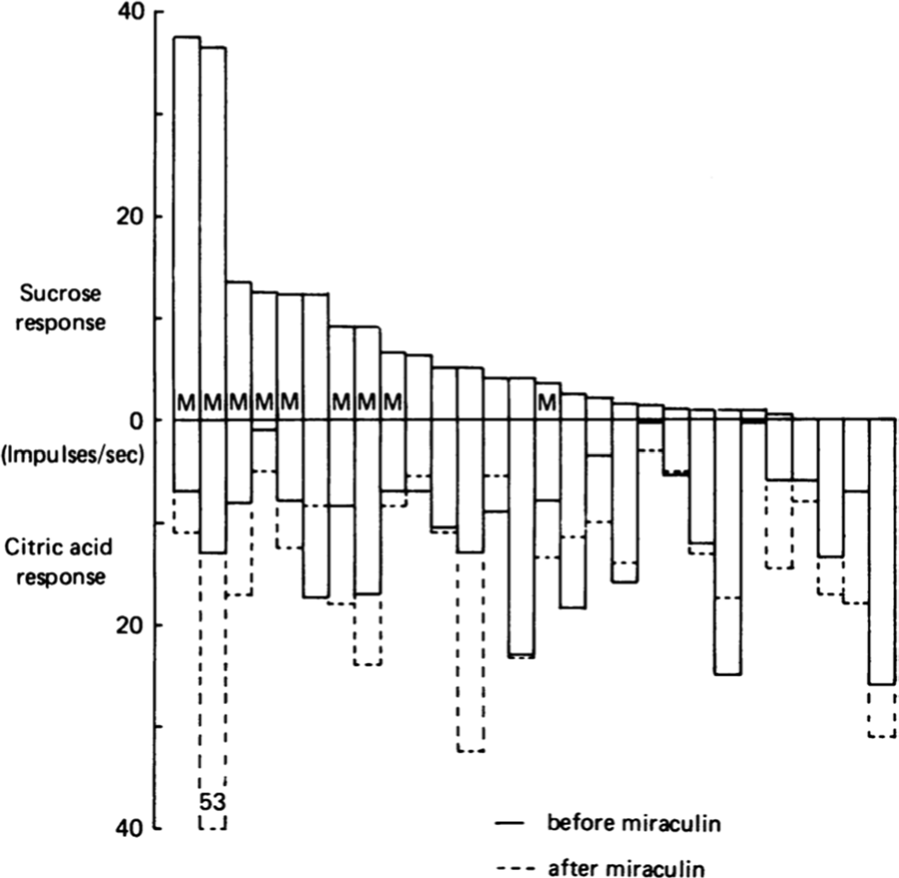
Responses to 0.3 M sucrose (plotted upwards) in S fibers and to 0.002 M citric acid in the same S fibers (plotted downwards) expressed as the number of impulses/secs during the first 5 s of stimulation. The dotted line in the columns show the responses to the citric acid in the same S fibers after miraculin. The response to citric acid was significantly increased in fibers labelled with M. In an acidic environment miraculin stimulates the sweet receptors on TRCs, which then transmits impulses in their S fibers. These data resolved the riddle on how miraculin works and added support to labelled-line hypothesis. [1]

Figure 22 shows responses to sucrose in CT S fibers (plotted upwards) and their response to 0.02 citric acid (plotted downwards) before miraculin was applied to the tongue. The broken columns show the same fibers’ response to citric acid after miraculin application. On the whole, S fibers increased their response to citric acid approximately two–fold after miraculin application. This explains the greatly increased intake of acid in Fig. 21**.** [1]. Figures 21, 22 show data that fulfill the second condition of the labelled-line theory in the sense that a new taste quality is created by S fiber activity after miraculin, namely sweet quality. The mechanism is the same as in marmoset; miraculin attaches to sweet receptors without stimulating these. This happens first when the pH is < 7 at which point miraculin acts as a stimulus. Our conclusion was confirmed more than 27 years later with different technique [63].

*In conclusion, the rhesus monkey behavioral responses to tastants parallel more closely that of human than marmoset because rhesus taste fibers in both CT and NG cluster more closely according to human taste qualities than in the marmoset. The finding of a strong representation of Q fibers in NG from the back of the tongue and N and S fibers in CT from the front, corresponds* with *the human distribution of mostly bitter taste on the back and sweet/salt on the front and makes it more suitable as a model of taste in human. In addition, miraculin stimulates S fibers when acids are used as stimuli., This coincides with an increase in acid consumption. We conclude that at pH* < *7, miraculin stimulates taste cells with sweet receptors, initiating impulses in synapsing S fibers, thereby adding a new taste quality*. *The data show that the taste fibers in rhesus monkey satisfy the condition for labelled-line.*

### Chimpanzee *Pan troglodytes*

This section summarizes behavioral, summated and single fiber results from 19 adult chimpanzees (*Pan troglodytes*) [34, 45, 51–57].

**Fig. 23:**
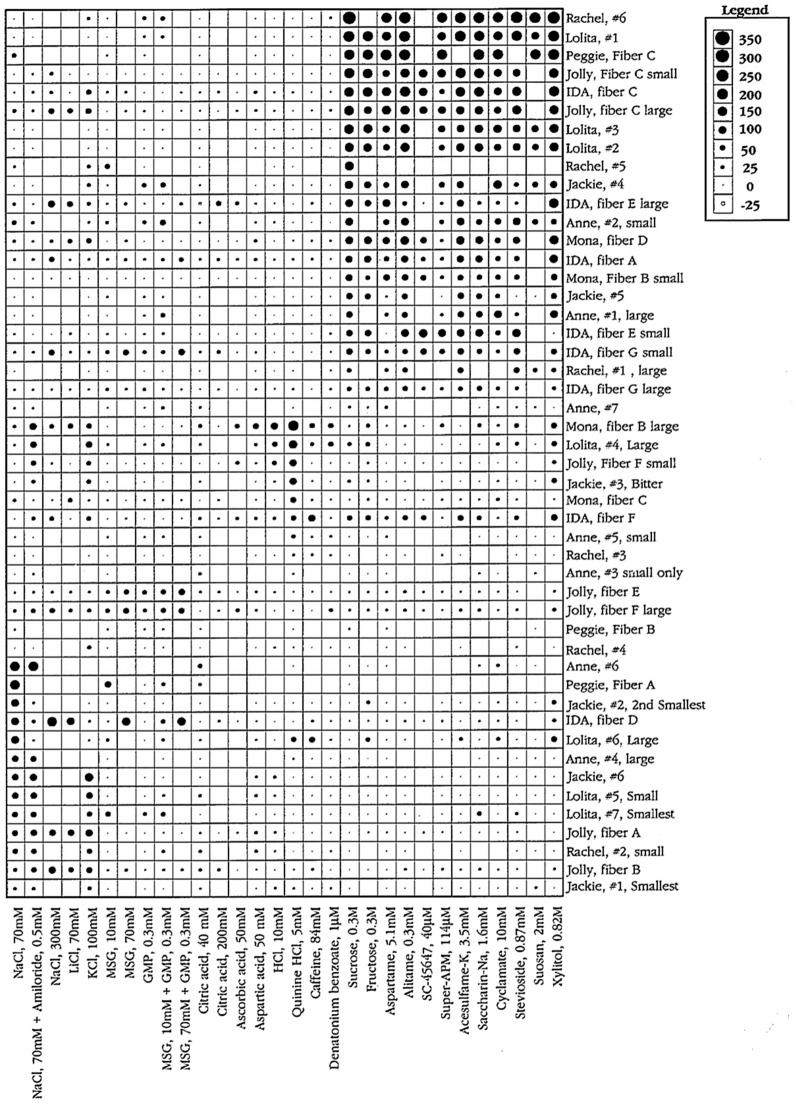
An overview of the results of 48 single fibers from the CT recorded in chimpanzee and presented in a topographical method. The area of the circles represents the number of impulses during the first 5 s of stimulation. Open circles represent inhibition or no response. The stimuli were arranged in the order of salt, sour, bitter, and sweet along the x axis. The fibers were arranged along the y axis in groups: NaCl-, acid-, QHCl-, and sucrose-best fibers. It is likely that the missing population of Q fibers is caused by the same factor as recorded in rhesus, namely bitter is better represented on the back of the tongue. We were never offered the opportunity to record from the NG in chimpanzees. [56]

Figure 23 shows that taste fibers in chimpanzee fall into human categories. The figure presents an overview of the response in 48 CT fibers. The topographical plot shows that the S fibers responded almost exclusively to the 12 sweeteners but not to the other non-sweet compounds. These sweeteners were all selected, because they are known sweeteners in human. The single fibers responding to QHCl formed a group with no overlap to the sweet compounds. This was also the case with the fibers responding to NaCl. It is important for the conclusions of this study that increased stimulus concentration had no effect on the taste quality of the taste fibers. Comparisons of the results with 70 and 300 mM NaCl and 40 and 200 mM citric acid gave the same result in this cluster analysis. Although 300 mM NaCl gave some response in some other fibers, it never stimulated as much as that fiber type’s best stimuli. The organization of these chimpanzee taste fibers are basically the same as for rhesus and marmoset, but stricter in taste qualities in the sense that a fiber only responds to stimuli belonging to one taste quality. For example, the S fibers responded only to sweet. This was also the case for the N fibers, which are divided in the sub-categories.

#### Hierarchical cluster analysis showed that chimpanzee taste fibers fell in human taste qualities

**Fig. 24:**
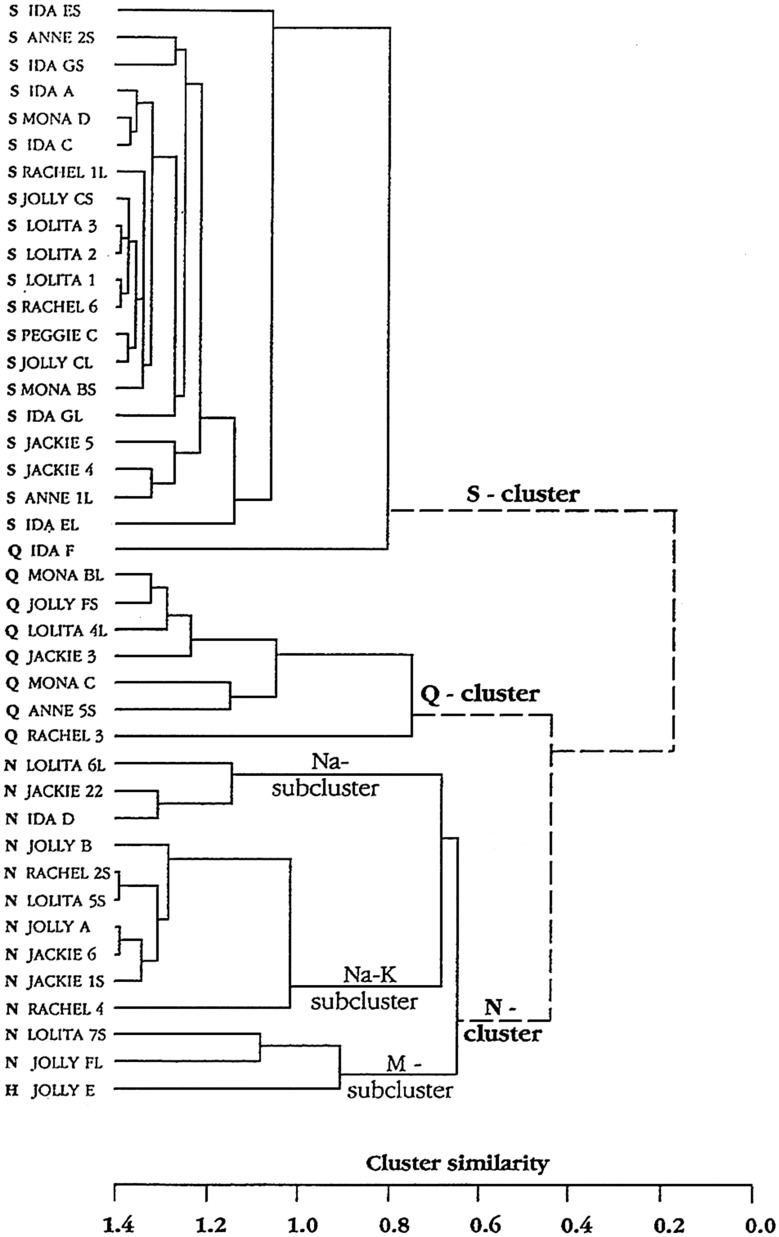
Hierarchical cluster analysis of the response profiles for 41 CT single fibers in chimpanzee. Inter cluster similarity was measured with the use of the Pearson correlation coefficient and cluster analysis proceeded according to the average linkage method. Fiber number and response category based on response to the 4 standard solutions of N, H, Q and S: NaCl-, citric acid-, QHCl-, and sucrose fibers, respectively. [56]

Figure 24 shows the result of hierarchical cluster analysis of the 41 fibers to 22 stimuli; the same fibers and stimuli used in Fig. 23. Analysis revealed 3 major clusters; a large S cluster, a small Q cluster and an N cluster divided into three subclusters.

In the following, we present each cluster of fibers. This division of N fibers into subgroups is interesting in the light of new knowledge of the possible existence of more than one salt receptor and transduction by [68]. Another interesting feature that parallels the findings in rhesus monkeys presented earlier, is that there are few Q fibers in the CT nerve of chimpanzee. It is likely that given the opportunity to record from NG of chimpanzee, we would have found a larger distribution of Q fibers compared to CT as we identified in rhesus monkey.

#### S-fiber cluster

**Fig. 25:**
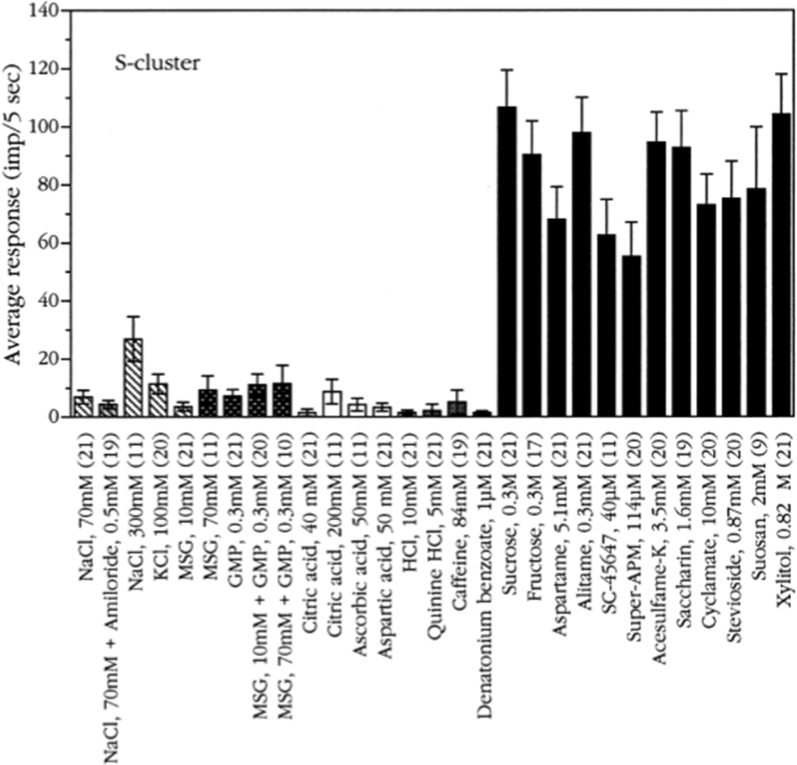
The average response profiles of the S-cluster. Error bars are SE. Striped columns indicate salts; dark grey columns, umami compounds; open columns, acids; grey columns, bitter compounds; and black columns, sweeteners. Numbers within brackets show number of fibers tested with each compound. The plot shows that chimpanzee S fibers are tuned to sweet because there was hardly any response to the non-sweeteners. [56]

Figure 25 shows the average of the responses in S fibers to different stimuli. The plot shows that in chimpanzee the only significant response in S fibers was caused by compounds sweet to humans, while all the other stimuli elicited insignificant fiber activity. Even 300 mM NaCl elicited less than 30 impulses per second which contrasts to the > 100 per second for sucrose. As mentioned in the introduction we adjusted the sweetener concentration to be about equal, based on human perceptions. The plot demonstrates how similar human and chimpanzee senses of taste are, because, for example, the amplitudes are about the same for all sweeteners.

**Fig. 26:**
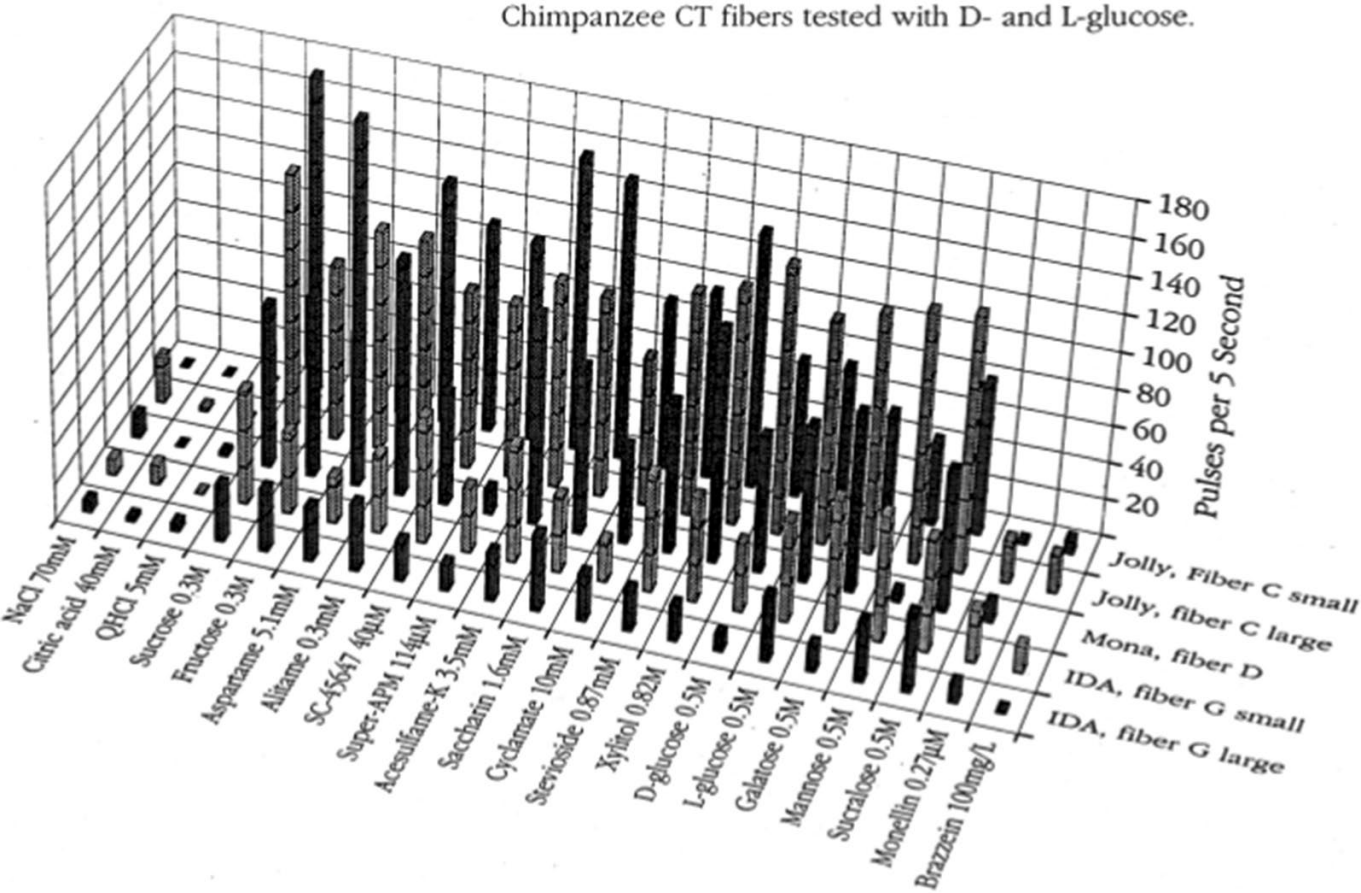
The results of stimulation of 5 sucrose-best fibers with an extended array of sweet compounds. The Figure shows that every sweetener stimulated the S fibers and that the responses were very uniform in each S fiber. [56]

Figure 26 shows the results of stimulation of 5 S fibers with an extended array of compounds sweet to humans. The plot shows that every S fiber responded to every sweetener. It is also remarkable how uniform the responses were within a fiber to the sweeteners. The array included sweeteners only sweet to humans but not sweet to non-primates or even South American primates, such as the sweet proteins brazzein and monellin, galactose, mannose and sucralose. The relative smaller responses of the sweet proteins brazzein and monellin, were partly the result of their slow onset of taste. With regard to the response to D- and L-glucose it is well known that chirality may play a role in taste. The difference in taste between D- and L- tryptophan serves as an example, as one is sweet and the other has no sweetness. However, humans taste no difference between D- and L-glucose [69]. The plot shows there was no difference in the glucose response magnitude observed in chimpanzee where the mean response to D-glucose of 67 imp/5 s, and 73 imp/5 s for L-glucose. Behavioral tests showed also no difference in chimpanzee consumption between D- and L-glucose (*p* = 0.5). In addition, there was no evident difference in the temporal profiles elicited by D- and L-glucose in the single fiber recordings (not shown). Notice also that there was essentially no S fiber response to NaCl, citric acid and QHCl. This shows how specific the S fibers are for sweeteners, a feature that supports further the labelled- line pattern of taste coding.

#### Q-fiber cluster

**Fig. 27:**
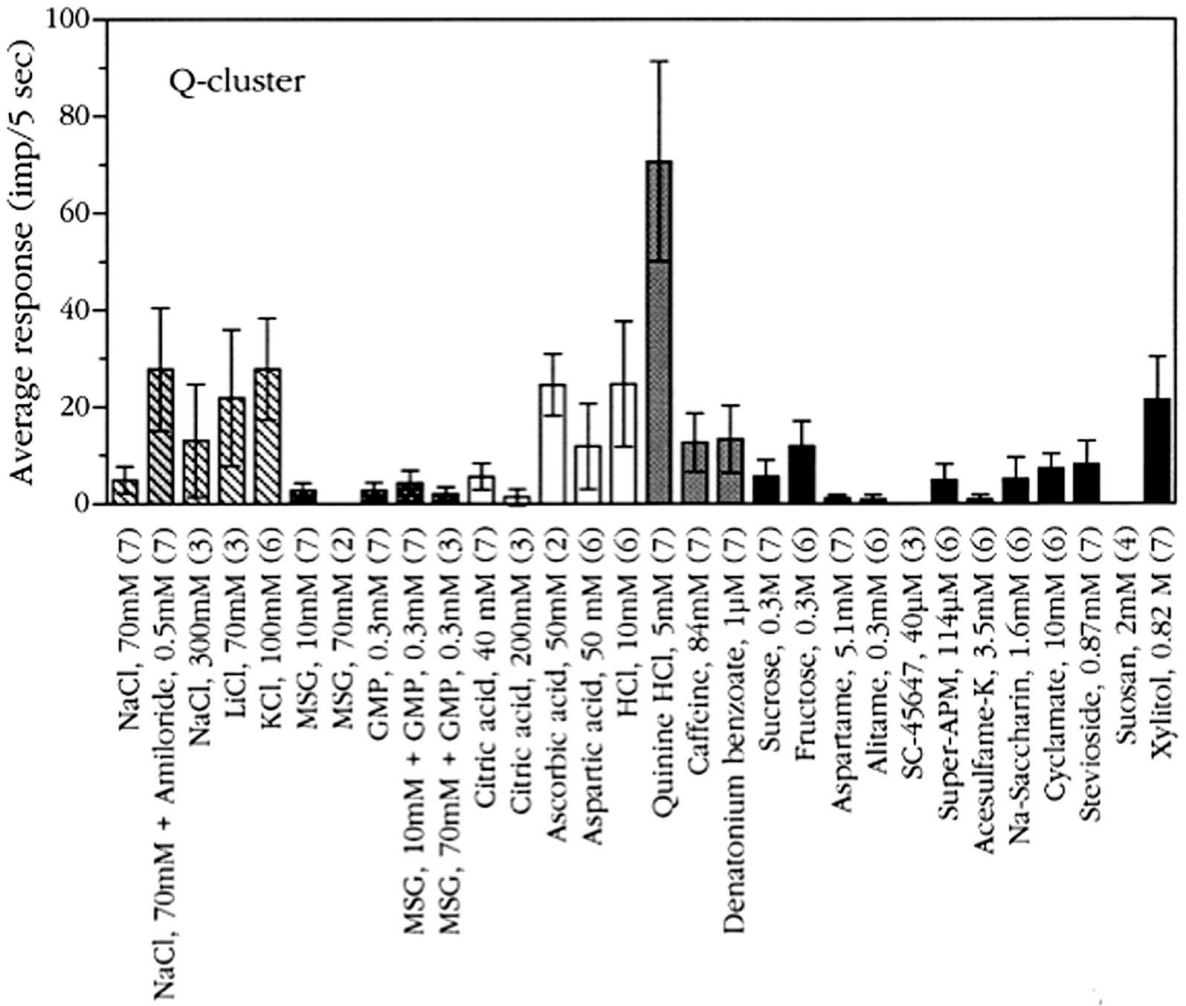
Averaged responses of Q-fibers in chimpanze. Bars illustrate SE. Different patterns of columns indicate different taste qualities. Striped columns indicate salts; dark grey columns, umami compounds; open columns acids; grey columns, bitter compounds; and black columns, sweeteners. Numbers within brackets show number of fibers tested with each compound. [56]

Figure 27 shows average responses of 7 Q fibers. Generally, Q fibers formed a less homogeneous category and were more broadly tuned than N and S fibers. QHCl was by far the most effective bitter stimulus, while caffeine, denatonium benzoate and KCl hardly elicited any responses. The responses to a mixture of NaCl and amiloride were larger than the responses to NaCl alone, suggesting that the addition of amiloride increased bitterness. This is also the experience in humans. Stimuli of other taste qualities, such as citric acid, elicited little activity or no activity, as did NaCl and the umami compounds. Sweeteners that to humans elicit a less ‘‘clean’’ sweet taste, such as xylitol, stevioside, cyclamate, and saccharin also stimulated some bitter fibers. This fits with the notion that their taste is less sucrose-like than, for example, that of aspartame, alitame, and acesulfame- [70]. However, it is likely that in chimpanzee, as in humans and rhesus, bitter taste is better represented by responses in NG.

#### N-fiber clusters

**Fig. 28:**
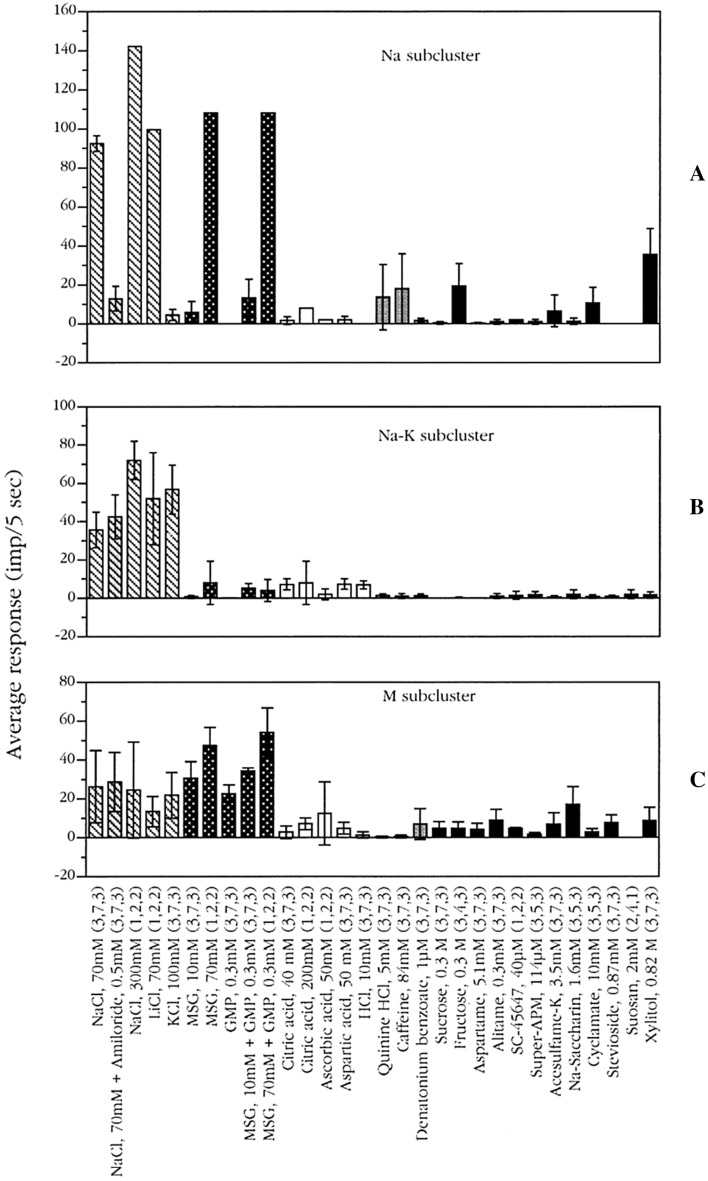
The average responses of the 3 subclusters: Na-subcluster, Na–K-subcluster, and M-subcluster. Striped columns indicate salts; dark grey columns, umami compounds; open columns, acids; grey columns, bitter compounds; and black columns, sweeteners. Error bars are SE. Numbers within brackets show number of fibers tested with each compound. [56]

Figure 28 shows that the 13 fibers in this cluster consisted of three subclusters: Na, Na–K, and M subcluster. Na fibers responded strongly to and were quite specific to NaCl and LiCl, but did not respond at all to KCl. This shows that the salty taste of KCl is very different to that of NaCl in chimpanzee as has been determined also in humans. Amiloride diminished the response to NaCl, which parallels psychophysical data in human [71, 72]. The second subcluster of Na–K fibers consisted of 5 fibers, for which KCl elicited as large of response as NaCl. Their response to NaCl was unaffected by the addition of amiloride. Finally, the M subcluster consisted of 3 fibers and responded best to MSG with or without GMP and equally to NaCl, LiCl and KCl. In all 3 fibers, the response to MSG mixed with GMP was larger than to the compounds alone. This supports the existence in chimpanzee, as has been deduced in human, of a separate umami taste quality. The close relationship between these subclusters and present knowledge of receptors for salty taste [68] will be discussed later.

#### Multidimensional scaling

**Fig. 29:**
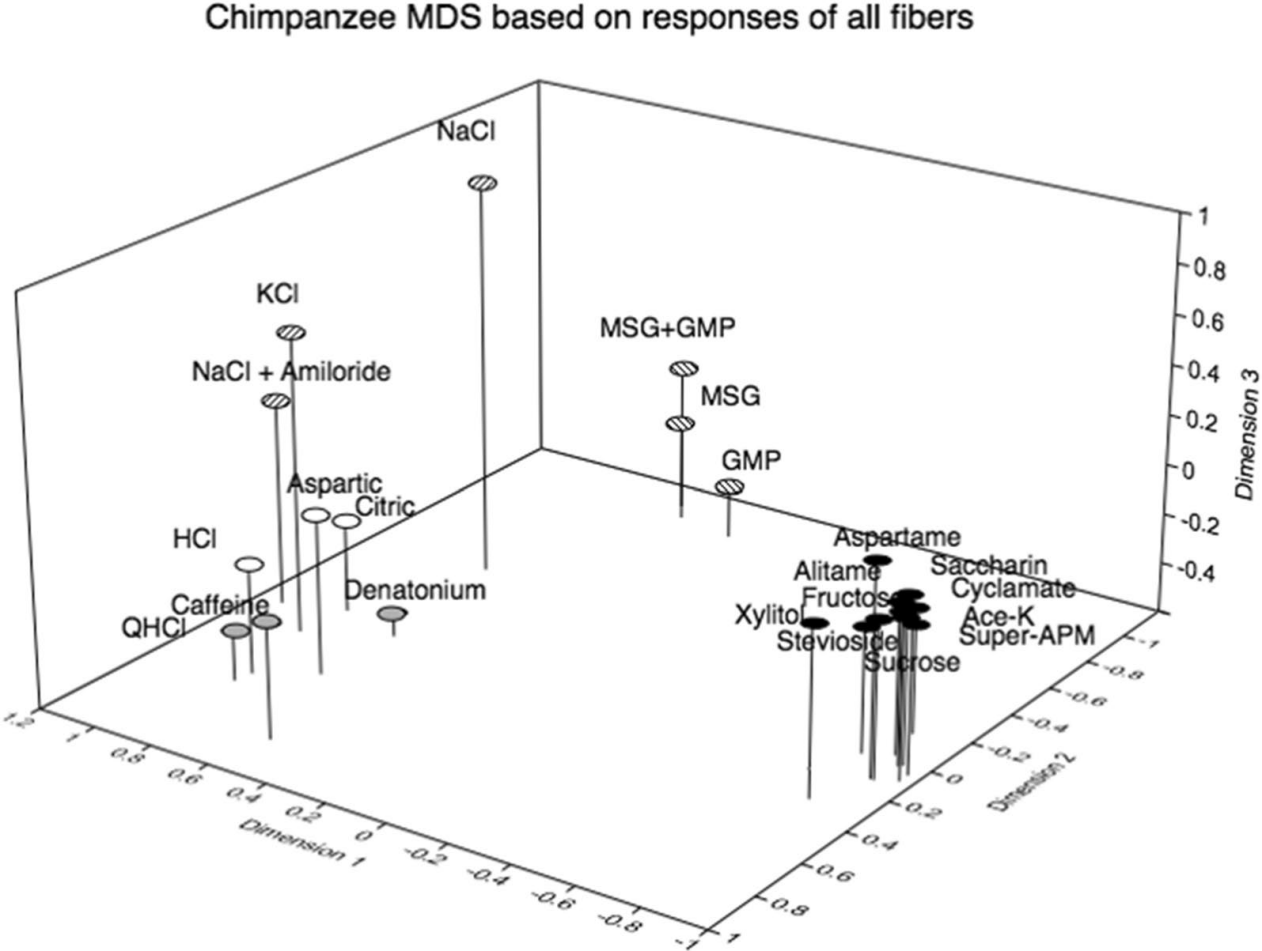
Distribution of 22 stimuli in a 3D space resulting from multidimensional scaling. Responses of all 48 CT fibers were used to generate a Pearson correlation matrix. Kruskal stress value is 0.049. The MDS analysis visualizes the level of similarity of the stimuli used based on the response in each fiber. Dimension 1 separated all the sweeteners in a very close group away from the other stimuli. Dimension 2 showed the umami tasting compounds as fairly close together but separate from sweet. Dimension 3 separated NaCl from the other salts and bitter stimuli, which included KCl and a mixture of NaCl and amiloride. Basically, conforming with human division into 5 taste qualities. [56]

Figure 29 is a 3D presentation of 22 stimuli of 48 CT nerve fibers. As mentioned earlier, MDS groups stimuli using the similarity of the taste fiber response they evoke. The first impression is that the compounds form tighter groups according to their taste quality than in rhesus and marmoset indicating that to chimpanzees the compounds representing each taste quality had a more similar taste than to rhesus and marmoset. In Fig. 29 dimension 1 separates all the sweeteners in a very tight group further from the other taste stimuli. Dimension 2 shows the umami tasting compounds as fairly close together, but still separate from sweeteners, and not similar to NaCl, which fits with human taste experiences. Dimension 3 separates NaCl from the other salts and bitter stimuli, which includes KCl and a mixture of NaCl and amiloride. This demonstrates that NaCl in itself has a unique taste to hominoid primates. This group of fibers provides a distinctive pattern of activity that can distinguish NaCl from all other compounds. The results clearly suggest that KCl tastes differently than NaCl and cannot be used as a taste replacement for NaCl. Generally, the MDS analysis showed a clearer grouping of bitter, salty, umami and sweet taste qualities in chimpanzee than in rhesus and marmoset. This is not surprising considering that all stimuli were selected according to their taste to humans and that chimpanzees are closest phylogenetically to humans.

#### Gymnemic acid effects the human sense of taste

**Fig. 30:**
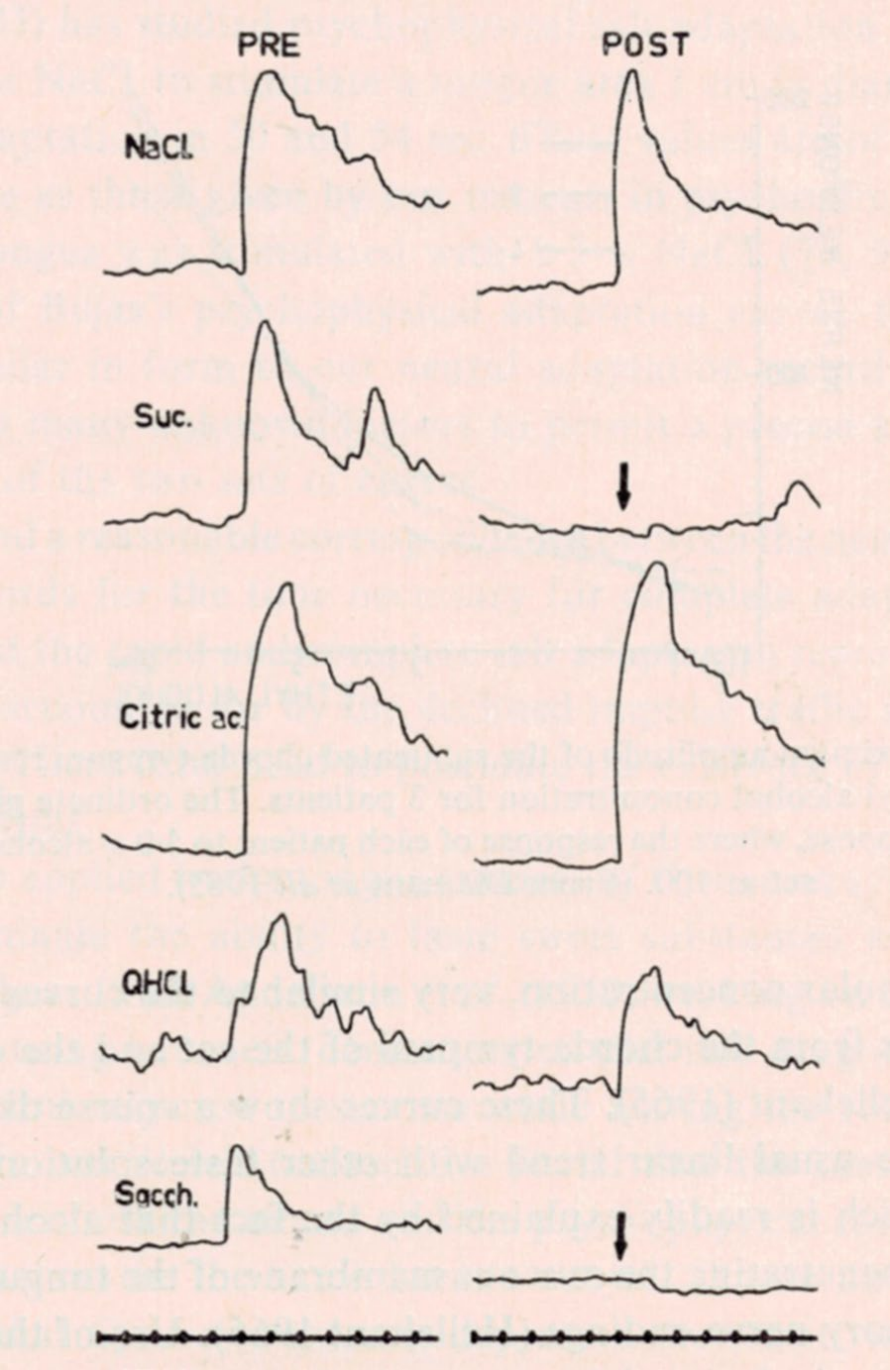
Summated recording from whole CT nerve in the human pre- and post-GA. Time marks in sec. The arrows show the onset of stimulation. [28]

The sweet blocking effect of Gymnemic acid (GA) in human has been known for centuries [73]. Figure 30 demonstrates that the human CT response to bitter, salty and sour stimuli are unaffected, but response to sucrose disappeared after GA oral exposure [28, 74]. We knew from earlier recordings that GA in non-primates and macaques does not block sweet [27, 30, 42, 51, 75, 76]. However, because chimpanzee is an Hominidae species, there were reasons to expect similar effect of GA on chimpanzee taste as in human. If GA blocks sweet taste in chimpanzee, it can be used to test Smith’s and Frank’s second condition for the labelled-line pattern of taste: blockage of activity in a particular cluster must cause blockage of one taste quality.

#### In chimpanzees GA lowers intake of human sweeteners

**Fig. 31:**
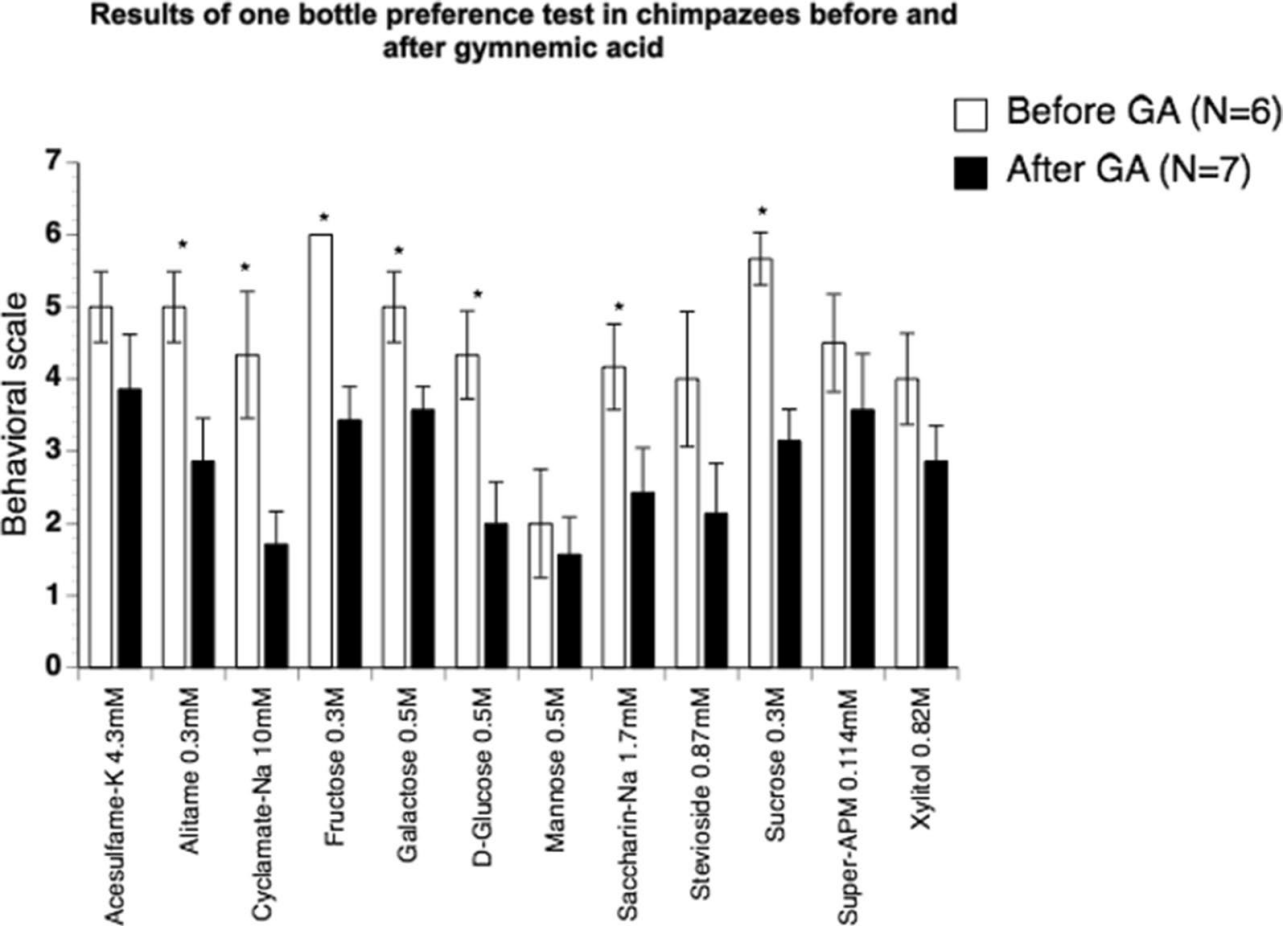
The columns show the results of the behavioral experiments before GA (white columns) and after exposure of whole mouth of adult chimpanzees to GA in ice-cream (black columns). We used the six-point behavioral scale to assess chimpanzees’ liking of solutions. The highest score (six points) was given if the animal, after tasting the solution, pulled the cup through the bar, licked it extensively, then shredded it so that the bottom of the cup could be licked, and finally kept the cup or what was left of it. Four points were given if the animal, upon tasting, pulled the cup into the cage, turned it upside down and licked it, but then dropped it. Two points were given if the animal consumed the mixture, pulled the cup into the cage, and finished the solution, but then lost interest. Zero points were given if the animal, after tasting the mixture, did not pull the cup into the cage and did not pay attention to the content. Intermediate points were given for behavior between these four main levels. The error bars show the SE. There are no error bars for fructose because all six animals got a score of 6. N signifies number of animals in each test. The asterisks mark the level of significance: p < 0.05. [34]

Figure 31 shows the intake of 12 equally sweet human sweeteners before GA (white columns) and after (black columns). We used a six-point scale to determine how attractive the solutions were both before and after GA. In both sessions animals were offered 10–12 ml of each of the sweeteners in paper cups. During the second session, when the effect of GA on the sweeteners was studied the animals were given the solutions with GA. Figure 31 shows that following GA application intake of 9 out of 12 sweeteners was significantly reduced. The suppressions were statistically significant for sucrose, fructose, alitame, cyclamate, galactose, glucose, and saccharin (p < 0.05), but the effect of GA was evident in the intake of all sweeteners c.f. [34].

#### Gymnemic acid abolishes the response to sweet in S fibers

**Fig. 32:**
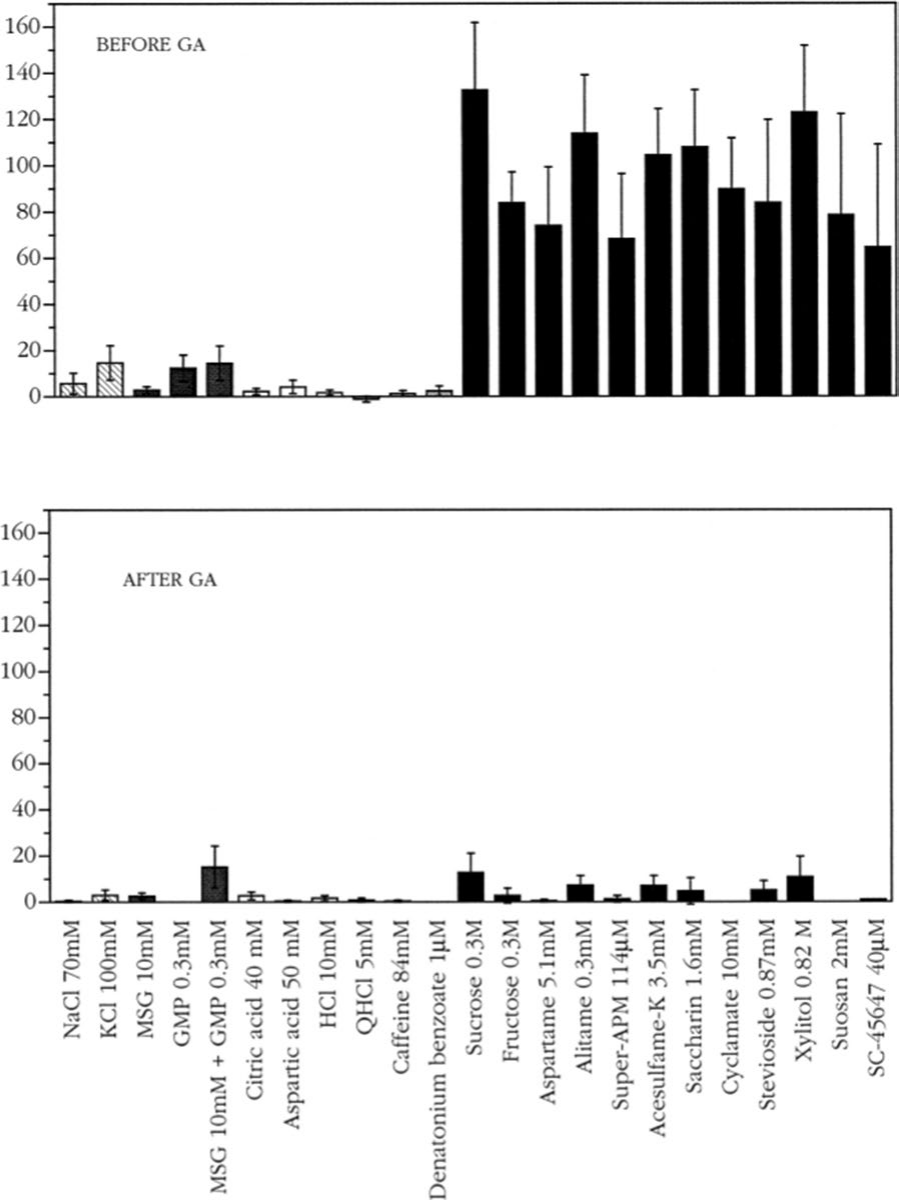
The average responses of the S fibers before and after application of GA in chimpanzee CT nerve. Data were averaged for six fibers. Error bars are SE. Key: hatched columns, salts; dark gray columns, umami compounds; open columns, acids; gray columns, bitter compounds; black columns, sweeteners [57]

Figure 32 presents averages of the responses of all S fibers recorded. The upper plot shows the responses before and the lower plots after the application of GA on the tongue. A comparison of the two plots shows that S-fiber response sweeteners is basically abolished by GA. It is also evident that S fibers were neither stimulated by non-sweet stimuli nor affected by GA. They responded only to sweet stimuli.

#### Gymnemic acid’s effects on Q and N fibers

**Fig. 33:**
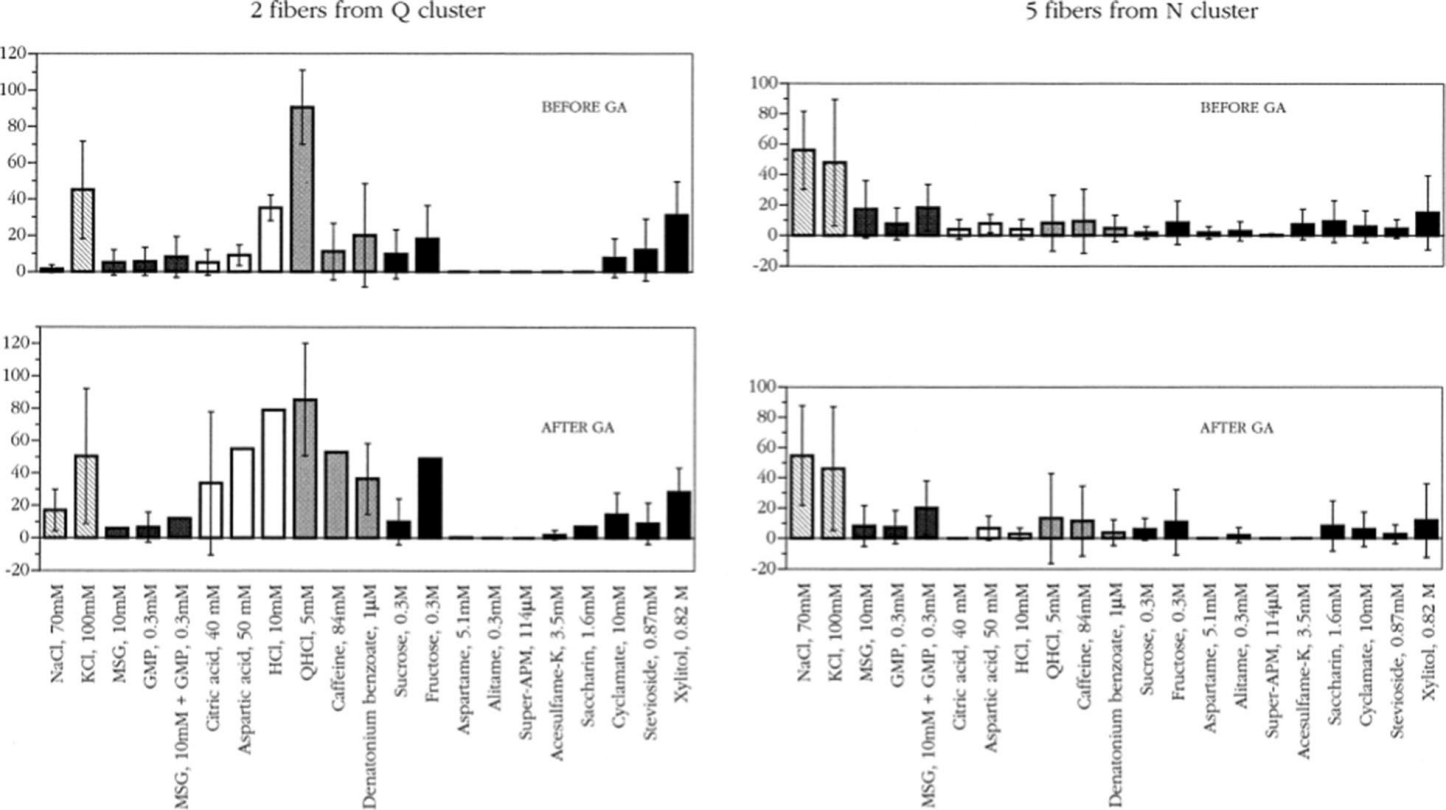
Effect of GA on responses of fibers that do not belong to the S-cluster in chipanzee CT. Graphs on the left side demonstrate the average response profiles of two Q-cluster fibers, and graphs on the right side demonstrate the average response profiles of five N-cluster fibers. Error bars are SE. Different patterns of columns indicate different taste qualities of the stimuli. [57]

Figure 33 presents averages of the responses of the fibers from Q and N clusters. The upper and lower plots show respectively the responses before and after the application of GA. It is evident that the responses of these fibers were unaffected by application of GA. Statistical analysis showed no difference between the responses before and after GA.

#### S, Q and N fibers tested individually with GA

**Fig. 34:**
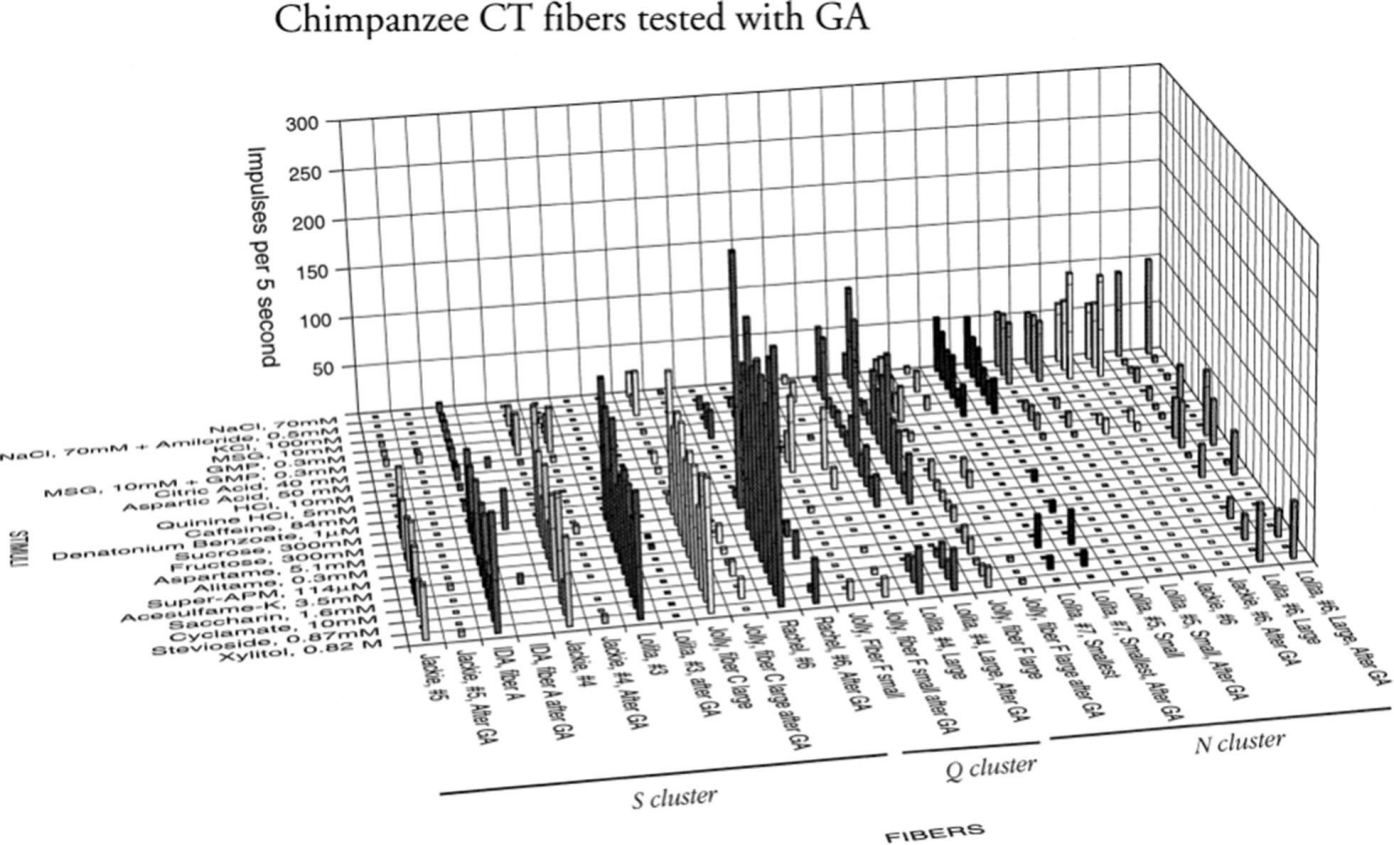
The plot summarizes the effect of GA in the chimpanzee. These recordings included 13 single chorda tympani fibers before and after application of GA on the tongue. The stimuli are arranged in the order of salt, sour, bitter, and sweet along the y-axis. Every second row labelled “after GA” shows data obtained after GA. The fibers are arranged along the x-axis in groups: S-, Q-, and N-clusters. Absence of a mark shows that data are missing. [57]

Figure 34 shows the result of GA on taste responses of single chorda tympani fibers presented individually. The *x-*axis shows the S-cluster (6 fibers), Q-cluster (2 fibers), and N-cluster (5 fibers) before and after GA. The tongue was first stimulated with the entire series of stimuli. Then the fungiform papillae innervated by the fiber were located with the tip of a fine point made of cotton soaked in the best stimulus for that particular taste fiber. The area usually included one to three fungiform papillae. After we had satisfactorily localized the fungiform papillae or papilla gymnemic acids in solution (2 mg/mL) was applied in a piece of filter paper cut to the size of the area. The paper was left on the tongue for a minimum of 3 min. The stimulation sequence, sometimes abbreviated, was then repeated. The method saved material and left the rest of the tongue available for further single fiber recordings without having to wait for the disappearance of the effects.

These data are important because it shows that GA removes only the sweet taste quality and exerts no effects on the bitter and salty taste qualities or any other non-sweet taste. For example, the very right recording is “N fiber Lotta #6 large”, which responded to all stimuli in the same manner before as after GA, while the response to sweet of the “S fiber Jackie #5” on the very left is absent after GA, but no other non-sweet taste stimuli are affected. It is equally important for the validity of the labelled-line coding hypothesis, that fibers not affected by GA respond to other taste qualities such as bitter or salty tastants. Both of these requirements are met in the chimpanzee.

*This satisfies also the second condition for labelled-line: blockage of activity in a particular cluster must cause blockage of one taste quality, but of no others.*

#### Miraculin effects on S fibers

In order to investigate how similar the effect of miraculin was on chimpanzee compared to macaques and marmosets, we recorded the effect of miraculin on a few chimpanzee CT fibers. It should be pointed out that time limitation of invasive procedures is an overwhelming concern in animals whose value cannot be assessed. Therefore, our miraculin data are limited in size. Nonetheless, the results are in complete accordance with our earlier findings on the effect of miraculin in monkey and marmoset.

**Fig. 35:**
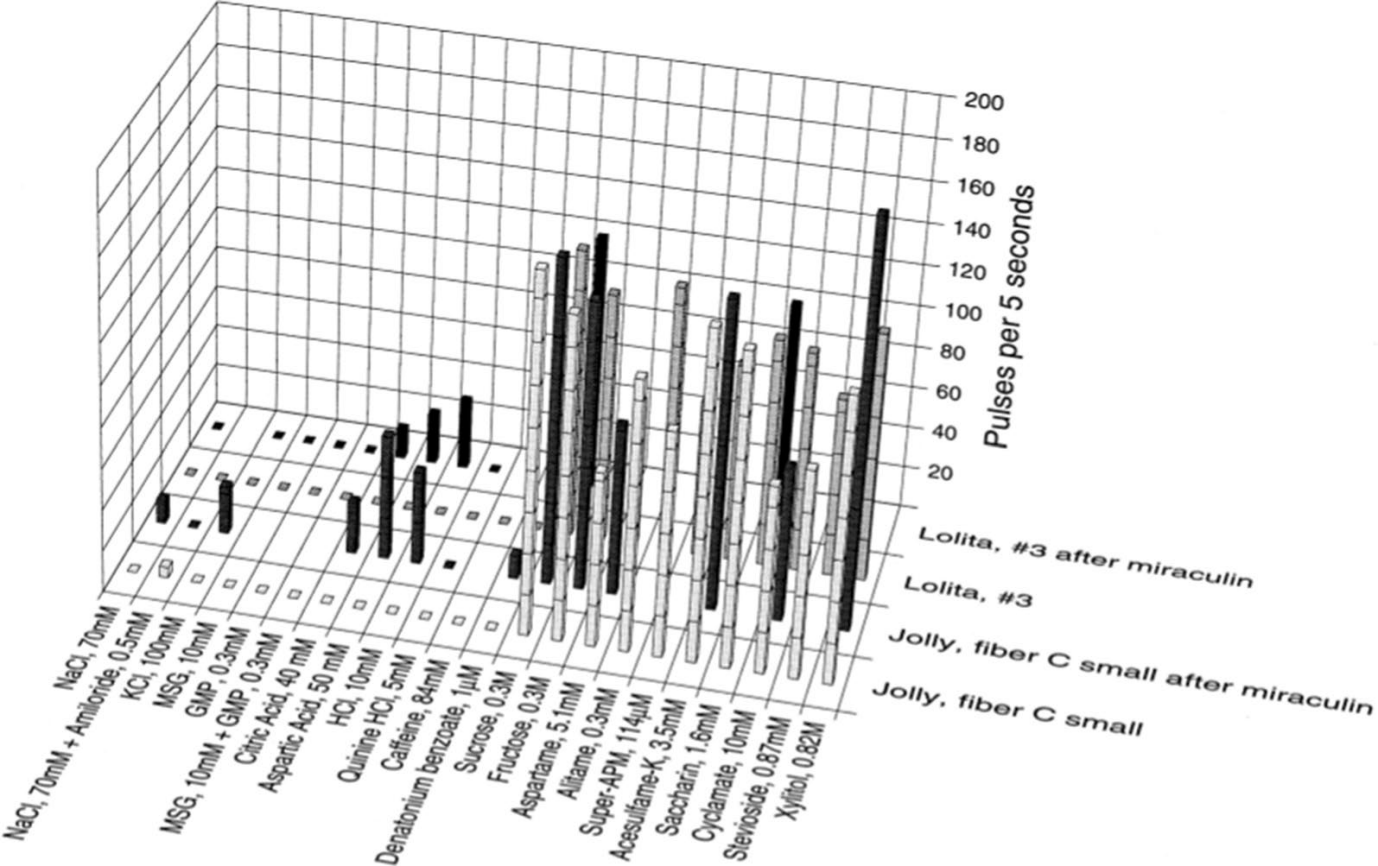
Effect of miraculin on responses of two S fibers in chimpanzee. The stimuli are arranged in the order of salt, sour, bitter, and sweet. Absence of a filled mark shows that data are missing. The front row of each set of plots shows the response in the fiber before miraculin. [57]

Figure 35 shows the result of application of miraculin in two S-fibers. The front row of each set of plots shows the response in the fiber before miraculin. As can be seen, both fibers responded exclusively to the sweeteners. After application of miraculin, citric acid, aspartic acid, and HCl elicited a response in each S fiber, fulfilling the second condition for support of the labelled-line pattern of taste coding postulated by Smith and Frank.

#### Miraculin and gymnemic acid on an S-fiber

**Fig. 36:**
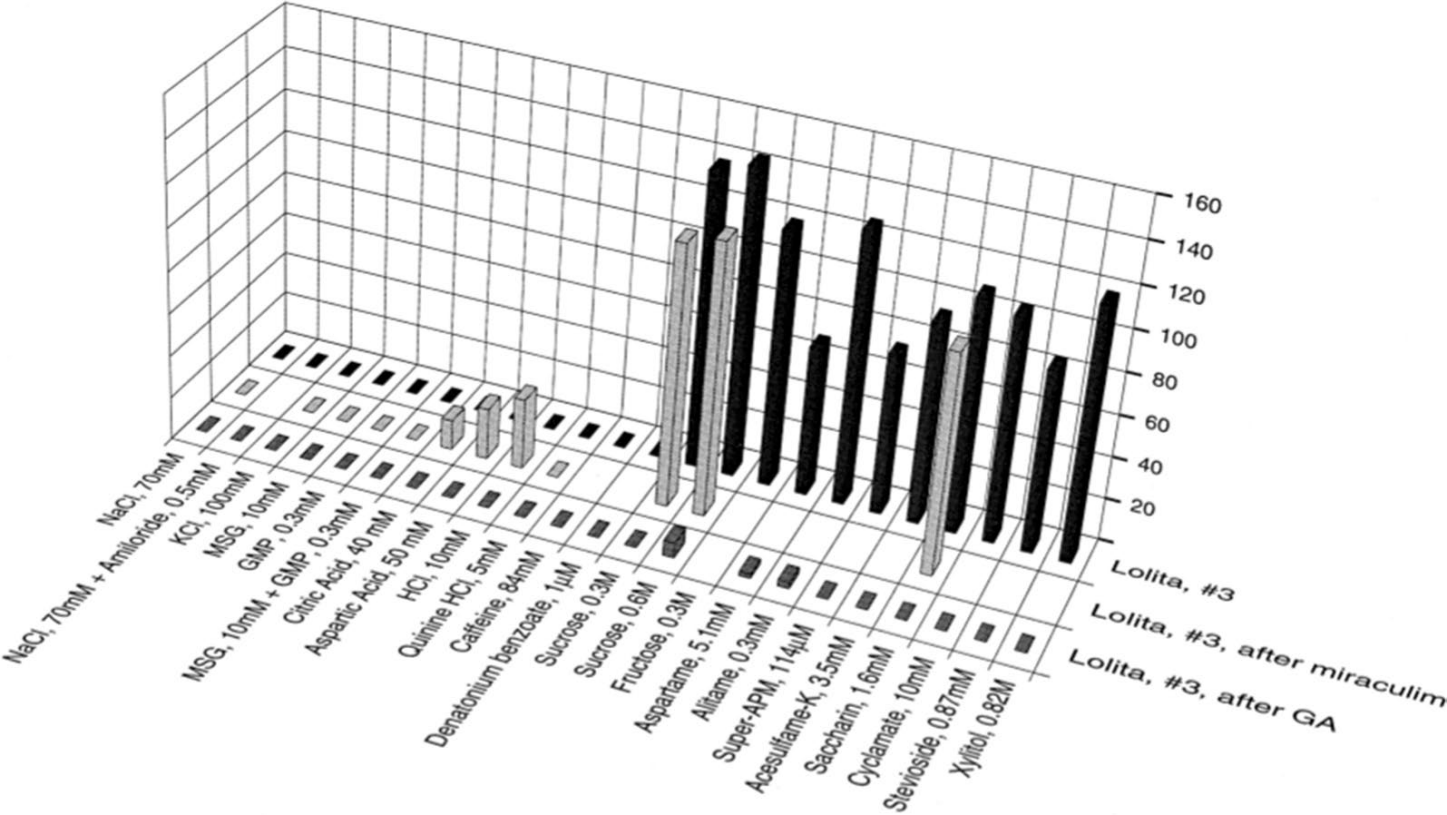
Responses of S-cluster fiber in chimpanzee before treatment (background row), after application of first miraculin (middle row), and then GA (front row). The stimuli are arranged in the order of salt, sour, bitter, and sweet. Absence of a filled mark shows that data are missing. [57]

In humans, GA application following miraculin abolishes the miraculin-induced sweetness of acids, as well as abolishes the sweetness of sweeteners [77]. In Fig. 36 we show first the effect of miraculin and then GA to one S fiber in chimpanzee. The back row shows the S fiber responses to 11 sweeteners before application of miraculin. The next row was obtained after application of miraculin. It was important to minimize the time between application of miraculin and GA so that time would influence as little as possible the observed effects of GA on miraculin-responses. Therefore, we limited the number of sweeteners but included most of the non-sweet compounds. As shown, the fiber responded to acids after miraculin, the sweeteners still elicited a response, and there was no response to other stimuli. The data represented in the front row was obtained after GA had been applied to the papilla previously exposed to miraculin. The fiber shows practically no response to either sweeteners or to acids. These data show that the application of GA abolished the response to both sweet and miraculin-induced response to sour stimuli in this S-fiber. The response to sucrose in the front row shows that the S fiber was physically alive. The results are in complete fulfillment of all conditions for the labelled-line coding in primate taste.

*In conclusion, we did not record any differences in the sense of taste between human and chimpanzee. The sweet taste receptor in chimpanzee is almost identical to the one in human* [78, 79]. *The sweet blocking effects of GA are the same as in human and all fibers in the sweet cluster ceased to respond to every sweetener after GA on the tongue. The miraculin effect in chimpanzee is identical to marmoset, rhesus and human, consisting of a S fibers response to acids and increased intake of acids after miraculin on the tongue. These results indicate that the chimpanzee is the ultimate model for elucidating human sense of taste.*

In the following we will discuss

- A. The influence of phylogeny on the sense of taste.
- B. Taste buds and labelled-line coding.
- C. Müller’s law of specific nerve energies and labelled-line coding.
- D. Labelled-line coding and higher brain center.
- E. More recent studies supporting labelled-line coding of taste.

#### On the influence of phylogeny on taste

**Fig. 37:**
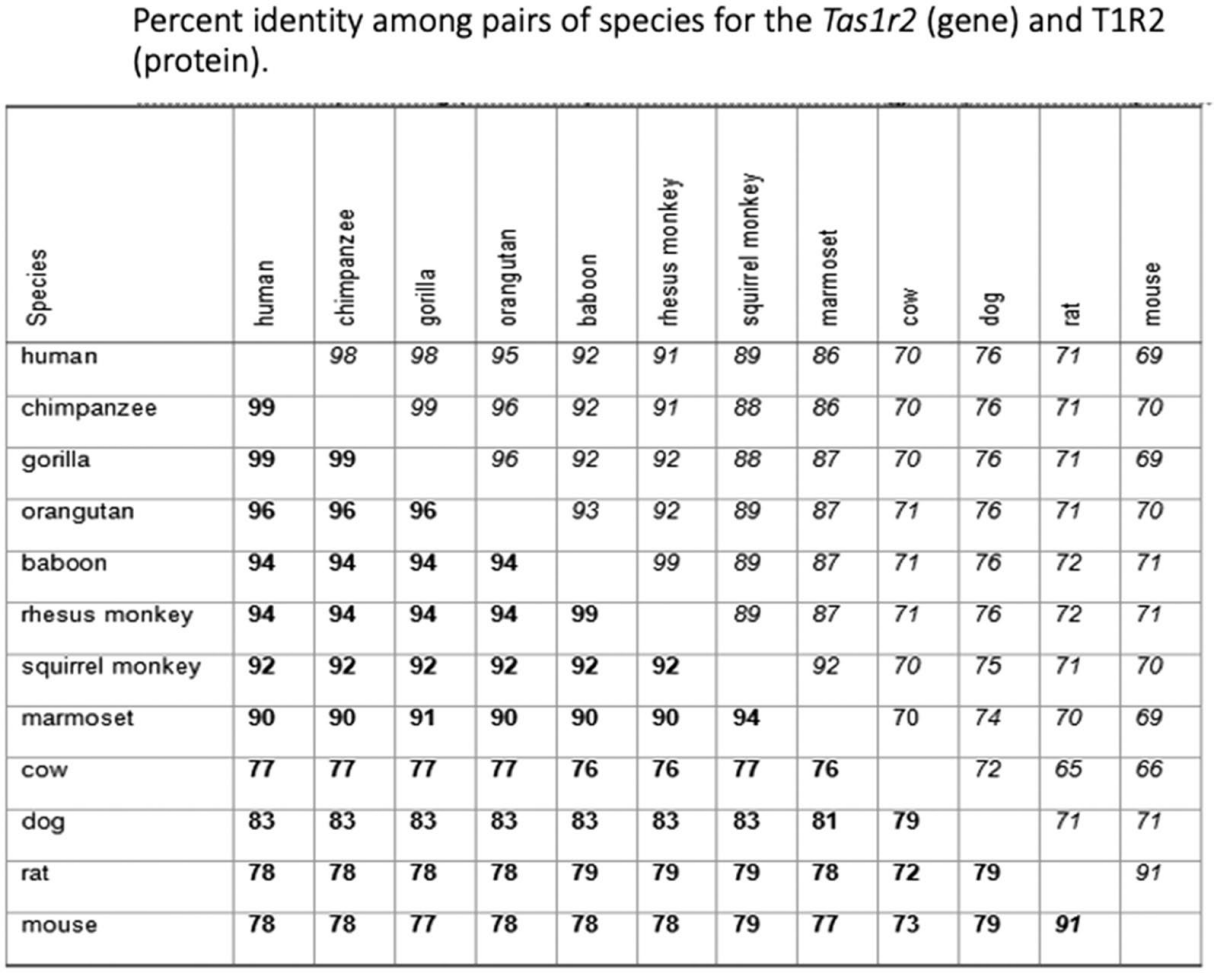
Percent identity among pairs of species for the Tas1r2 gene and T1R2 protein for a number of primate and non-primates [79]

Figure 1 presented several examples of phylogenetic influence on the sense of sweet taste in primates**. **Figure 37 shows how the Tas1r2 gene and protein T1R2 (part of the sweet receptor T1R2/R3 discovered in 2000 [80] [81]) differ between humans and a number of primates and non-primates [78, 79]. The differences between human and chimpanzee proteins and genes are small, between 1 and 2%. But the differences increase with distance between species. Thus, the gene/protein differences between humans and orangutans is 4%, 6% in baboons and in rhesus monkeys, and 10% in South American marmosets. It is evident that this can be traced to phylogeny.

**Fig. 38:**
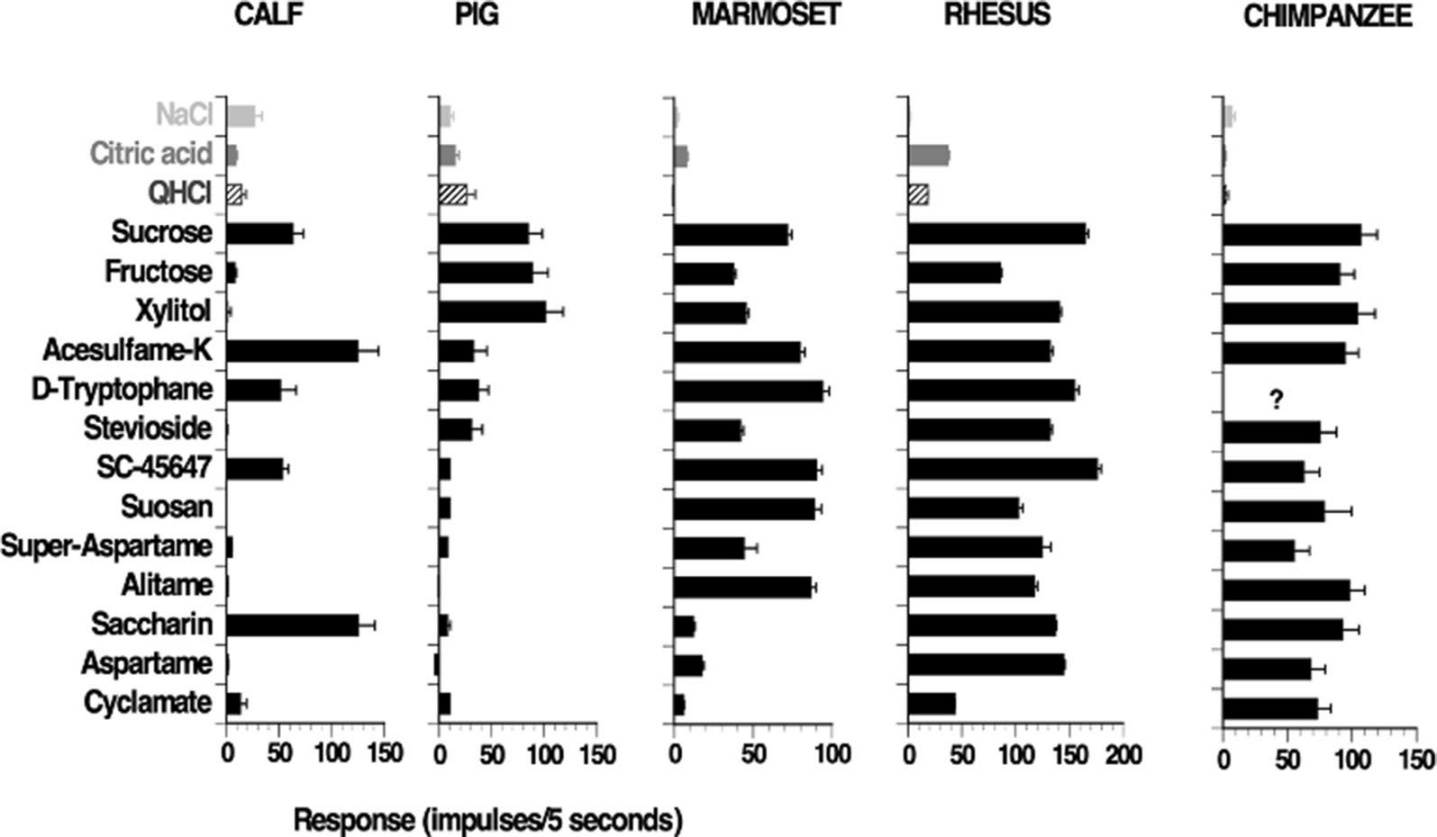
This figure shows the influence of species variation in S fiber responses of a numer of species to compounds which to human have approximately the same sweetness. The number of S fibers was; in chimpanzee 20, rhesus monkey 14, pig 16, and hamster 12. The top bar in each graph shows the S fibers’ response NaCl, citric acid, and QHCl. [55]

Figure 38 presents another example of the phylogenetic influence on S fiber responses to 13 sweeteners, which were approximately equally sweet to humans. The graph shows that in chimpanzee all these sweeteners elicited similar S fibers responses, but with a larger variation in rhesus monkeys and even greater in marmoset. In pig and calf the majority of the sweeteners elicited no S fiber response at all. These results may explain that phylogenic diversity of the animals used in taste research influence the ability to solve the taste code. The fact is that this was pointed out early [82–85].

#### Taste buds and labelled-line coding

Taste receptor cells (TRCs) in the taste buds are constantly turning over and new ones will only differentiate when taste fibers reach the TRC [86–88]. Signals are exchanged between the taste fibers and TRCs. Consequently, the handshaking between TRCs and taste fibers is an ongoing process in which the nerve fibers continuously try to identify suitable taste cells and develop the right connection [89]. This explains the extensive number of nerve fibers in and around TBs. This would also explain why Finger et al. [90] reported that 71% of taste cells and Ryba et al. [89] reported 80% of taste cells had a taste fiber connection. This is a very high percentage of the TRCs with taste fiber connection if one considers the turn-over rate of TRCs [86].

One condition of labelled line coding is that each TRC carries only one kind of receptors. The function of a labelled-line system requires correct linkage between the receptor, TRC fiber and cortical area of taste quality. A second condition for the labelled-line coding organization to be valid is that each taste fiber synapses with a TRC carrying the receptor compatible with the cortical taste area it is carrying its message to, for example a TRC with T1R2/R3 to the cortical taste area giving a sweet taste sensation. The conclusion above was obtained with a different technique than ours, proves that our conclusion that taste functions as a labelled-line is correct.

#### Müller’s law of specific nerve energies and labelled-line coding

From the conclusion that taste is coded as labelled-lines in single nerve fibers, it follows that Müller's law of "specific nerve energies" [91] also applies to taste. In 1912 Lord Adrian [92] confirmed that Müller’s “specific nerve energies” [91] are action potentials in nerve fibers. Studies have shown the validity of Müller’s law for other somatic senses such as nociceptive, mechanical and thermal senses [93], vision [2] and audition. It makes no difference for the sensation, for example in the case of audition, if the receptor in the cochlea or its sensory fiber is stimulated mechanically or electrically, or in any other manner, provided the action potential reaches the auditory area of the cortex. This is also the case with taste and the gustatory cortex. The study shows that taste is the last to join the other somatosensory system such as vision [2] and touch [93]. Taste qualities are defined by the pathway in which the taste receptor information is carried as action potentials to the part of the brain where the nerve impulses give rise to one or the other of the 4–5 taste qualities.

#### More recent studies supporting labelled-line coding of taste

Some 20 years after our labelled-line conclusion in primates, Lee et al. presented an elegant confirmation of the above conditions and the existence of labelled-line taste coding in mice [89]. They engineered mice with S fibers that linked to TRCs with bitter receptors and Q fibers linked to TRCs with sweet receptors. They reported that the engineered mice consumed and preferred bitter solutions over sweet solutions. They concluded that:” That together, these results uncover the basic logic of the wiring of the taste system at the periphery and illustrate how a labelled-line sensory circuit preserves signaling integrity despite rapid and stochastic turnover of receptor cells” [89]. Taste signals are relayed by multiple brain regions, including the parabrachial nucleus (PBN) of the pons, before reaching the gustatory cortex via the gustatory thalamus. Further support for our findings that each taste quality has its own receptors and neuronal pathway is presented by Fu et al. [94] who demonstrated that SatB2 neurons in the mouse parabrachial nucleus encode positive valence and selectively transmit sweet signals to the gustatory thalamus. Fu et al. concluded that taste information from the periphery is encoded in a labelled-line manner. Their results are completely in line with our conclusions in higher non-human primates.

Information in the taste fibers passes through several synapses in brain stem, which allows for influences from other areas of the CNS and the digestive system, but the information will eventually end in the cortical taste area which is divided into distinct cortical fields where the action potentials of each fiber cluster give rise to a taste quality [95–97].

## Conclusion

The taste from the TBs is transported in single nerve fibers as action potentials. In higher primates, single taste fibers cluster according to the human division of taste qualities. Similar to other sensory systems, taste travels as action potentials in labelled-lines, according to Müller’s law of specific nerve energies, through the brain stem and appropriate areas of CNS to cortex where each taste quality is created. Importantly, what is recognized as the human taste qualities sweet, sour, bitter, salt and umami, are relevant in higher primates, mostly so in the chimpanzee, but becomes less relevant with the species phylogenetically more distant to human.

The taste system is similar to ones used by other sensory systems mediating other sensory modalities such as vision, pain, and thermal and mechanical stimuli. One clinical consequence of labelled-line coding in taste is that it may be possible to trace the cause of ageusia or hypogeusia to the periphery by recording the action potentials in human taste fibers because the CT crosses the middle ear close to the tympanic membrane.

## Data Availability

The raw data referred to in this Review was acquired before 2000 in USA and are no longer available.

## Abbreviations

TB: Taste buds
TRC: Taste receptor cell
GA: Extract from *Gymnema sylvestre*
Mir: Miraculin
CT: Chorda tympani proper nerve carrying action potentials from TB
NG: The lingual part of the glossopharyngeal nerve carrying action potentials from TB
